# Interplay of Ser273 Phosphorylation and K268 and K293 Acetylation in PPARγ: Implications for PPARγ Activation

**DOI:** 10.1002/prot.70140

**Published:** 2026-04-12

**Authors:** Caique Camargo Malospirito, Marieli Mariano Gonçalves Dias, Gabriel Ernesto Jara, Thayná Mendonça Avelino, Giovanna Blazutti Elias, Paulo Sergio Lopes de Oliveira, Ana Carolina Migliorini Figueira

**Affiliations:** ^1^ Brazilian Center for Research in Energy and Materials (CNPEM), Brazilian Biosciences National Laboratory (LNBio) Campinas Brazil; ^2^ Graduate Program in Molecular and Morpho Functional Biology Institute of Biology, State University of Campinas (Unicamp) Campinas Brazil; ^3^ Graduate Program in Pharmaceutical Sciences, Faculty of Pharmaceutical Sciences State University of Campinas (Unicamp) Campinas Brazil

## Abstract

Post‐translational modifications (PTMs) play a critical role in regulating the transcriptional activity of PPARγ, a nuclear receptor central to glucose and lipid homeostasis. Among these, lysine acetylation at K268 and K293 and phosphorylation at S273 are particularly relevant to insulin sensitivity. These residues form a regulatory binding interface for protein partners such as SIRT1 and CDK5, which exert opposing effects on PPARγ activity—SIRT1 promoting deacetylation and insulin sensitization, and CDK5 driving phosphorylation linked to insulin resistance. Here we show that modifications at this interface influence PPARγ's interaction with its regulators and its transcriptional activity. Acetyl‐mimetic mutations at K268 and K293 reduce CDK5 binding and phosphorylation, while enhancing transcriptional activity. Phosphorylation at S273 weakens SIRT1 binding and limits its repressive function, even under overexpression. These effects likely reflect both direct interference with protein docking and changes in the global acetylation or phosphorylation landscape. Our findings reveal that this PTM‐rich interface functions as a regulatory hub, integrating signals from multiple protein partners to fine‐tune PPARγ activity. Unlike full receptor activation by agonists, which often triggers adverse effects, modulating this interface represents a refined therapeutic avenue for enhancing insulin sensitivity in metabolic diseases like obesity and type 2 diabetes, with improved specificity and reduced side effects.

## 1 Introduction

The peroxisome proliferator‐activated receptor gamma (PPARγ) is a nuclear receptor that regulates the expression of genes involved in metabolic homeostasis [1, 2]. Its key role in metabolic regulation made PPARγ a molecular target for developing ligands to treat metabolic disorders, such as insulin resistance, a hallmark feature of the metabolic syndrome [3, 4]. The thiazolidinediones are a family of agonist drugs that target PPARγ and improve glucose homeostasis in insulin‐resistant patients. However, the administration of rosiglitazone has been associated with several adverse effects beyond those intended ones. Therefore, novel strategies to modulate PPARγ's activation toward beneficial metabolic outcomes while minimizing adverse effects of strong agonists like rosiglitazone are crucial [5].

PPARγ functions as a transcription factor that modulates the expression of genes involved in metabolic regulation. Its transcriptional activity is tightly controlled by corepressor and coactivator proteins, which respectively inhibit or enhance its function. The dynamic exchange of these coregulators is a critical step in toggling PPARγ transcriptional activity on or off. Agonist drugs such as rosiglitazone modulate PPARγ activity by stabilizing its structure in an active conformation, thereby promoting the recruitment of coactivator proteins to the transcriptional complex [6]. The ligand‐binding domain (LBD) of PPARγ plays a central role in this regulatory process, as it contains the binding pocket for ligands such as rosiglitazone and fatty acids, as well as key sites for post‐translational modifications (PTMs) that collectively influence PPARγ activation. PPARγ undergoes multiple PTMs—including SUMOylation, ubiquitination, acetylation, O‐GlcNAcylation, and phosphorylation—which occur at specific amino acid residues and modulate its functional state [7]. Among these, phosphorylation at serine 273 (S273p) and acetylation/deacetylation at lysines 268 and 293 (K268ac and K293ac) are particularly relevant due to their established roles in insulin resistance. Therefore, investigating these PTMs is crucial, as they may offer alternative strategies for modulating PPARγ activity and developing targeted therapies aimed at improving insulin sensitivity in metabolic‐related disorders.

PPARγ plays a central role in the pathophysiology of metabolic syndrome, linking obesity to insulin resistance through its regulation of lipid metabolism and adipocyte gene expression [8]. These alterations in gene expression patterns are associated with phosphorylation at serine 273 (S273p), which results from elevated activity of the kinase CDK5 and subsequently drives coregulatory protein exchange [6, 9, 10, 11]. Several PPARγ ligands have been shown to inhibit S273 phosphorylation, thereby counteracting its deleterious effects by promoting insulin sensitivity and improving lipid metabolism [11]. Consequently, targeting S273p represents a promising therapeutic strategy for the treatment of insulin resistance.

In addition to phosphorylation, the regulation of PPARγ acetylation also plays a key role in modulating its activity and insulin sensitivity. This balance is maintained by the histone acetyltransferase p300 and the NAD^+^‐dependent deacetylase SIRT1, which add and remove acetyl groups from lysine residues, respectively [7, 12]. Because p300 uses acetyl‐CoA and SIRT1 requires NAD^+^, both enzymes connect the metabolic state of the cell to the control of PPARγ function and downstream gene expression. In the context of insulin resistance, a shift toward deacetylation has emerged as a key regulatory mechanism controlling PPARγ activity. Specifically, SIRT1‐mediated deacetylation of acetyl‐lysines 268 (K268ac) and 293 (K293ac) modulates the transcriptional output of PPARγ, promoting an insulin‐sensitive phenotype. This modification drives white adipocytes toward a more energy‐expending state, enhancing the expression of genes involved in insulin responsiveness and contributing to improved metabolic function [13, 14, 15, 16, 17, 18, 19]. Thus, while S273 phosphorylation promotes insulin resistance, the deacetylation of K268 and K293 counteracts these effects by fostering an insulin‐sensitizing phenotype. Together, the regulation of these PTMs highlights a promising avenue for modulating PPARγ activity in metabolic disorders.

The PPARγ PTMs at S273p and at K268ac and K293ac appear to be closely interconnected in the regulation of its activity. Physiological and molecular evidence suggests that these PTMs do not act in isolation but rather influence one another in shaping PPARγ's transcriptional output [12, 20, 21]. For instance, studies have shown that deletion of SIRT1 specifically in adipocytes results in hyperacetylation of PPARγ at K268 and K293, while simultaneously reducing phosphorylation at S273. This shift is associated with enhanced insulin sensitivity and improved metabolic function [20], indicating that SIRT1‐mediated deacetylation may indirectly control S273p, possibly by altering the structural or functional state of PPARγ in a way that affects CDK5 accessibility or activity. Moreover, the constitutive acetylation of K268 and K293 has been experimentally linked to modulation of S273p, as demonstrated in [18, 19], reinforcing the hypothesis of a mechanistic crosstalk between these modifications. Such interplay implies that manipulating one PTM may influence the occurrence or impact of the other, creating an integrated regulatory network that fine‐tunes PPARγ's function in response to cellular metabolic cues. These findings underscore the importance of considering the combined effects of PTMs rather than evaluating them individually, particularly when designing therapeutic strategies aimed at improving insulin sensitivity through targeted modulation of PPARγ.

In this study, we investigate the interplay between Ser273 phosphorylation and K268/K293 acetylation in regulating PPARγ activation. By combining in vitro and in silico approaches, we provide structural and functional insights into how these PTMs influence PPARγ's interaction with the regulatory proteins SIRT1 and CDK5. Our findings demonstrate that these modifications not only alter the binding affinities of SIRT1 and CDK5 to PPARγ but also affect its transcriptional activity and the downstream consequences of SIRT1 and CDK5 overexpression. Together, these results underscore the functional crosstalk between phosphorylation and acetylation in modulating PPARγ and support PTM‐targeted interventions as a promising strategy for fine‐tuning its activity in metabolic disease contexts.

## 2 Material and Method

### 2.1 Clones and Protein Production

Plasmids used for transactivation assays in mammalian cells included pBIND‐PPARγ, which encodes a chimeric protein composed of the Gal4 DNA‐binding domain (DBD) fused to the PPARγ LBD; GRE‐LUC, containing the GAL4 sequence upstream of a firefly luciferase reporter gene; pRL‐TK, expressing *Renilla reniformis* luciferase as a transfection control; and Sirt‐pECE, used for mammalian SIRT1 expression (all obtained from Addgene, #1791) [22]. The cDNA‐CDK5 plasmid, encoding both CDK5 and its activator P35, was kindly provided by Professor Sang K. Park (Pohang University of Science and Technology). In addition, the p300‐pCI vector was a generous gift from Dr. Joan Boyes's group [23], and the pET28a‐SMT3‐SIRT1‐143CS plasmid for bacterial expression was donated by Rui‐Ming Xu (Chinese Academy of Sciences, National Laboratory of Biomacromolecules) [24]. Derivatives of PPARγ used in cellular and in vitro assays were generated by site‐directed mutagenesis following the QuickChange protocol (Agilent). Expression and purification of His‐tagged wild‐type and mutant PPARγ proteins were performed in *E. coli* BL21 (DE3) and purified as previously described in [12]. SIRT1‐CS expression and purification were carried out according to [12], and CDK5 was expressed and purified as described in [21].

### 2.2 Fluorescence Polarization Assay

The Dissociation constants (*K*_D_) of SIRT1:PPARγ, SIRT1:PPARγ (S273D) and CDK5:PPARγ (WT, K268Q, and K293Q) complexes were determined by fluorescence polarization (FP) assays, as described in [12, 21]. SIRT1 and CDK5 were labeled with FITC (fluorescein isothiocyanate) at a molar ratio of 3 μM FITC to 1 μM of SIRT1 and CDK5 in alkaline buffer (50 mM HEPES, pH 8.0, 100 mM NaCl), to ensure the deprotection of primary amines, allowing for covalent attachment of FITC to lysine residues and the N‐terminus of SIRT1. The labeling process took place at 4°C for 3 h, and the excess probe was removed using a desalting column (HiTrap, 5 mL, GE). Then, FITC‐labeled SIRT1 (80 nM) or CDK5 (20 nM) was incubated with increasing concentrations of the PPARγ LBD construct (ranging from 0.02 to 25 μM) in assay buffer (50 mM HEPES, pH 8.0; 100 mM NaCl; 1 mM DTT) in 384‐well plates (Greiner). A 20 min incubation at room temperature for CDK5:PPARγ and 4 h for SIRT1:PPARγ were done prior plate reading. The FP values were measured using a CLARIOStar plate reader (BMG LABTECH) with excitation and emission wavelengths of 495 and 520 nm, respectively. Fluorescence anisotropy measurements were performed using a ClarioStar plate reader (BMG) with an emission wavelength of 520 nm and an excitation wavelength of 495 nm. The *K*_D_ was determined by fitting the FP values to the Hill1 equation using OriginPro 8.6 software. Statistical analysis was performed using GraphPad Prism ANOVA with the Tukey test. *p*‐values: *p ≤ 0.05, **p ≤ 0.01, ***p ≤ 0.001, ****p ≤ 0.0001.

### 2.3 In Vitro Acetylation and Phosphorylation Assay

The acetylation levels of PPARγ were measured through an in vitro acetylation assay using the p300 Inhibitor Screening Assay Kit (Biovision) following the manufacturing protocol with the following modifications: For the reaction, 10 μM of PPARγ LBD WT and S273D mutant were used in quadruplicate following the manufacturer protocol. We assigned a negative control for each sample without acetyl‐CoA to exclude fluorescent bias from the intrinsic fluorescence of the proteins. Fluorescence values were obtained using CLARIOStar (BMG LABTECH) with an excitation/emission wavelength of 392 nm/482 nm in a 384‐white plate (Greiner). The acetylation levels for the samples were determined from the difference between the fluorescence values with and without acetyl‐CoA. Final fluorescence values were normalized for native PPARγ LBD construct, corresponding to 100% acetylation. Statistical analysis was performed using GraphPad Prism, a *t*‐test with *p*‐values: *p ≤ 0.05, **p ≤ 0.01, ***p ≤ 0.001, ****p ≤ 0.0001.

The levels of PPARγ phosphorylation (WT, K268Q, and K293Q) by CDK5 were measured by luminescent detection of ADP produced in the in vitro phosphorylation reaction with ADP‐Glo kinase assay (Promega) Kit, as in [21]. For this, 30 μM of purified PPARγ LBD construct was incubated with 25 ng of CDK5/p25 complex, kinase assay reaction buffer (Signal Chem III kinase assay buffer), and 30 μM ATP, for a final reaction volume of 5 μL. After the initial kinase reaction incubation, ADP‐Glo Reagent was added, and the reaction was incubated at room temperature for 40 min. The samples were then denatured at 95°C for 3 min. Then, the Kinase Detection Reagent was added, and the samples were incubated at room temperature for 30 min. The luminescence signal was measured on the ClarioStar plate reader (BMG Labtech). Statistical analysis was performed using GraphPad Prism, ANOVA with the Tukey test. *p*‐values: *p ≤ 0.05, **p ≤ 0.01, ***p ≤ 0.001, ****p ≤ 0.0001.

### 2.4 Cell Culture and Cellular Transactivation Assay

293 T cells (ATCC CRL‐3216), from human embryonic kidneys, were cultured in complete DMEM (Dulbecco's Modified Eagle's Medium) medium, containing 10% Fetal Bovine Serum (FBS) and 1% antibiotic (penicillin and streptomycin), and 0.37% sodium bicarbonate and kept at 37°C and 5% CO_2_. Cellular transactivation assays were performed to evaluate differences in PPARγ activation resulting from the presence and/or action of other proteins such as SIRT1, p300, and CDK5. We used PPARγ LBD clones linked to GAL4 DBD and its derivatives (K293Q, K293Q); the plasmid containing the GRE (GAL4 responsive element) fused to the firefly luciferase gene; and a transfection control vector that constitutively expresses luciferase from *Renilla reniformis* (pRL‐TK); in addition, clones of p300‐PCI, Sirt‐pECE, and CDK5‐pcDNA were used.

The 293 T cells were plated in a 24‐well plate (NEST) at a density of 1 × 10^5^ cells per well with 500 μL of complete DMEM medium; 24 h after plating, the medium was changed to incomplete DMEM (without fetal bovine serum or antibiotics), and the transfection mix was added, containing 400 ng of each plasmid and the JetPEI transfection reagent (Polyplus) in a 3:1 ratio (PEI:DNA) diluted in 150 mM NaCl. 4 h after the transfection, 1 μM rosiglitazone or 1 μM of DMSO was added, depending on the experimental condition [6].

The assay reading was performed 24 h after ligand treatment. The culture medium was added, and the cells were lysed with lysis solution from the Dual‐Luciferase Reporter Assay System (Promega) kit for 30 min in a shaker. The plate reader GloMax‐Multi Detection System (Promega) performed the luminescence reading. Luciferase production was measured using the Dual‐Luciferase Reporter Assay System (Promega) kit. Statistical analysis was performed using GraphPad Prism, ANOVA with the Tukey test. *p*‐values: *p ≤ 0.05, **p ≤ 0.01, ***p ≤ 0.001, ****p ≤ 0.0001.

### 2.5 Molecular Dynamics

The starting model for CDK5/PPARy was taken from reference [21]. The Amber ff14SB force field was used to describe the protein and the acetylated lysines. The ATP parameters were derived from Meagher et al. [25], and the parameters for Mg^2+^ were obtained according to [26]. We employed the TIP3P water model for solvation to create a truncated octahedral box with a 15 Å radius around the proteins [27]. Subsequently, the system was neutralized, achieving a salt concentration of 0.15 M by adding Na^+^ and Cl^−^ ions, utilizing the parameters outlined in [21, 28]. To obtain a relaxed and equilibrated structural model of the CDK5–PPARγ complex with ATP and Mg^2+^ in catalytically relevant coordination, we adapted a previously published model 21. The model was protonated at pH equal to 7.0 using H++ server [28, 29, 30]. The ATP conformation, coordinating water molecule, and Mg^2+^ were taken from the CDK2 structure (PDB ID: 1QMZ) after backbone alignment with CDK5. Initial energy minimization was performed with restraints of 100 kcal/mol/Å^2^ on backbone atoms (Cα, C, N, O), ATP, Mg^2+^, and the coordinating water (2500 steps steepest descent + 2500 steps conjugate gradient), followed by a second minimization under the same conditions but applied only to ATP, Mg^2+^, and the coordinating water. The minimized systems were used as a reference for the analysis.

The system was then equilibrated by MD simulations: 200 ps under NVT and 0.5 ns under NPT conditions, both with positional restraints of 100 kcal/mol/Å^2^ on ATP, Mg^2+^, and the coordinating water. After equilibration, Mg^2+^ coordination was refined by a 2 ns NPT simulation. During the first 500 ps, distance restraints were gradually increased (1 → 200 kcal/mol/Å^2^) between Mg^2+^ and the carbonyl oxygens of Asn131/Asn144 and the O3B atom of ATP. In the following 1.5 ns, these restraints were maintained at 200 kcal/mol/Å^2^. Additionally, throughout the 2 ns simulation, the distances OγSer273–PγATP and OγSer273–OGAsp126 were restrained with a force constant of 50 kcal/mol/Å^2^, while the OγSer273–PγATP–O3B angle was restrained to ≤°140° with a force constant of 50 kcal/mol/°^2^. Acetylated CDK5–PPARγ complexes (CDK5–PPARγK268Ac and PPARγK293Ac) were generated from the equilibrated CDK5–PPARγ model by replacing the residue name LYS with ALY in the PDB file prior to hydrogen addition. Missing atoms and hydrogens were subsequently added using tleap from AmberTools23. All systems were energy‐minimized without restraints using 10 steps of steepest descent followed by 990 steps of conjugate gradient.

Three independent 1 μs production MD simulations were carried out following the protocol described below: equilibration was performed in three stages: (1) the systems were heated from 50 to 300 K over 20 ps under NVT conditions with restraints of 5 kcal/mol/Å^2^ applied to Cα atoms; (2) a 2 ns NPT equilibration at 300 K was performed with restraints on backbone atoms (Cα, N, C), where the restraint force constant was gradually reduced from 4 to 1 kcal/mol/Å^2^ in steps of 0.5 ns; and (3) a final 1 ns NPT equilibration at 300 K without restraints. Production simulations of 1000 ns were then carried out for all models. Throughout all simulations, temperature was controlled using the stochastic Berendsen thermostat with a coupling constant of 1 ps, and pressure was maintained at 1 bar using a Monte Carlo barostat with a relaxation time of 1 ps. A 9 Å cutoff was applied for nonbonded interactions. The SHAKE algorithm was used to constrain all bonds involving hydrogen atoms, and an integration timestep of 2 fs was employed [31].

The SIRT1:PPARγ models used in this work were adapted from the models mentioned in reference [12]. These structures were used as a reference for SIRT1:PPARγ analysis. For SIRT1:PPARγ MDs, three 1 μs production MD replicas were run following the protocol below: we first applied an energy minimization of the solvent and side chains by restraining the protein backbone with a force constant of 100 kcal/mol. This process involved 10 steps of the steepest descent, followed by 990 steps of the conjugate gradient. Then, a minimization of the entire system was conducted without any constraints. The equilibration was carried out in seven steps. First, the system was heated from 25 to 298 K over 20 ps at NVT with constraints on the CA atoms using a force constant of 5 kcal/mol/Å^2^. The four NPT simulations of 10 ps each were performed, constraining the CA atoms with a decreasing force constant from 4 kcal/mol/Å^2^ to 1 kcal/mol/Å^2^. Then, a 1 ns NPT simulation without constraints, and, finally, a 1000 ns NPT production run at 298 K. The temperature was controlled using a Langevin thermostat [32] with a collision frequency of 1 ps^−1^. In comparison, the pressure was set to 1 bar using a Berendsen barostat with a relaxation time of 1 ps. All MD simulations were carried out using the PMEMD.cuda program from the AMBER22 package.

In addition to simulation analysis, we performed MM/PBSA calculations using a multiple‐trajectory approach. To isolate the energetic effect of acetylation from large‐scale conformational changes observed in the long MD simulations, we ran 25 independent short replicas (4 ns each) for the complexes, starting from the minimized initial structures. For the unbound state, only one independent simulation was generated for each component. The minimized structures were used as the starting point for all 25 MD replicas included in the MM/PBSA analysis. We used frames every 10 ps, resulting in 400 frames for each simulation used for MM/PBSA calculations. A total of 10 000 frames per system was used for MM/PBSA calculations. The ionic concentration was 0.15 M. Various internal dielectric constants were tested: 1, 2, 4, 6, and 8. All other options were set to default. The MPI version of MMPBSA.py [33] from AmberTools23 [34] was used for free energy calculations using the MM/PBSA approximation. Before producing replicas, the entire system was minimized without constraints. The process of generating replicas per complex involved seven steps: (1) the system was heated from 25 K to 298 K over 20 ps at NVT with constraints on the CA atoms using a force constant of 5 kcal/mol/Å^2^, (2–5) four NPT simulations of 10 ps each, constraining the CA atoms with a decreasing force constant from 4 kcal/mol/Å^2^ to 1 kcal/mol/Å^2^, (6) a 1 ns NPT simulation without constraints, and (7) a 3 ns NPT production run at 298 K. The MMPBSA free energy for the 25 replicas for each system was analyzed by a bootstrap resampling using boot, a R library, using 100 000 bootstrap trials [32]. The root mean squared (RMSD) analysis, the distance of the center of mass between proteins and the distance and angle for the nucleophilic attack of Ser273‐OG to ATP were performed using cpptraj from AmberTools23 [33, 34]. Plots were created using ggplot2 [35], a library of R [36], and RStudio [37]. The interfacial contacts were measured by calculating the Q score (fraction of native contacts) [38], using as a reference the minimized structure of each system. MDAnalysis was used for Q calculation by setting a cut‐off of 5 Å, a β of 5 Å, and a λ constant of 1.8 Å [39].

## 3 Results

### 3.1 K268ac, S273p and K293ac Are in the SIRT1 and CDK5 Docking Sites

**FIGURE 1:**
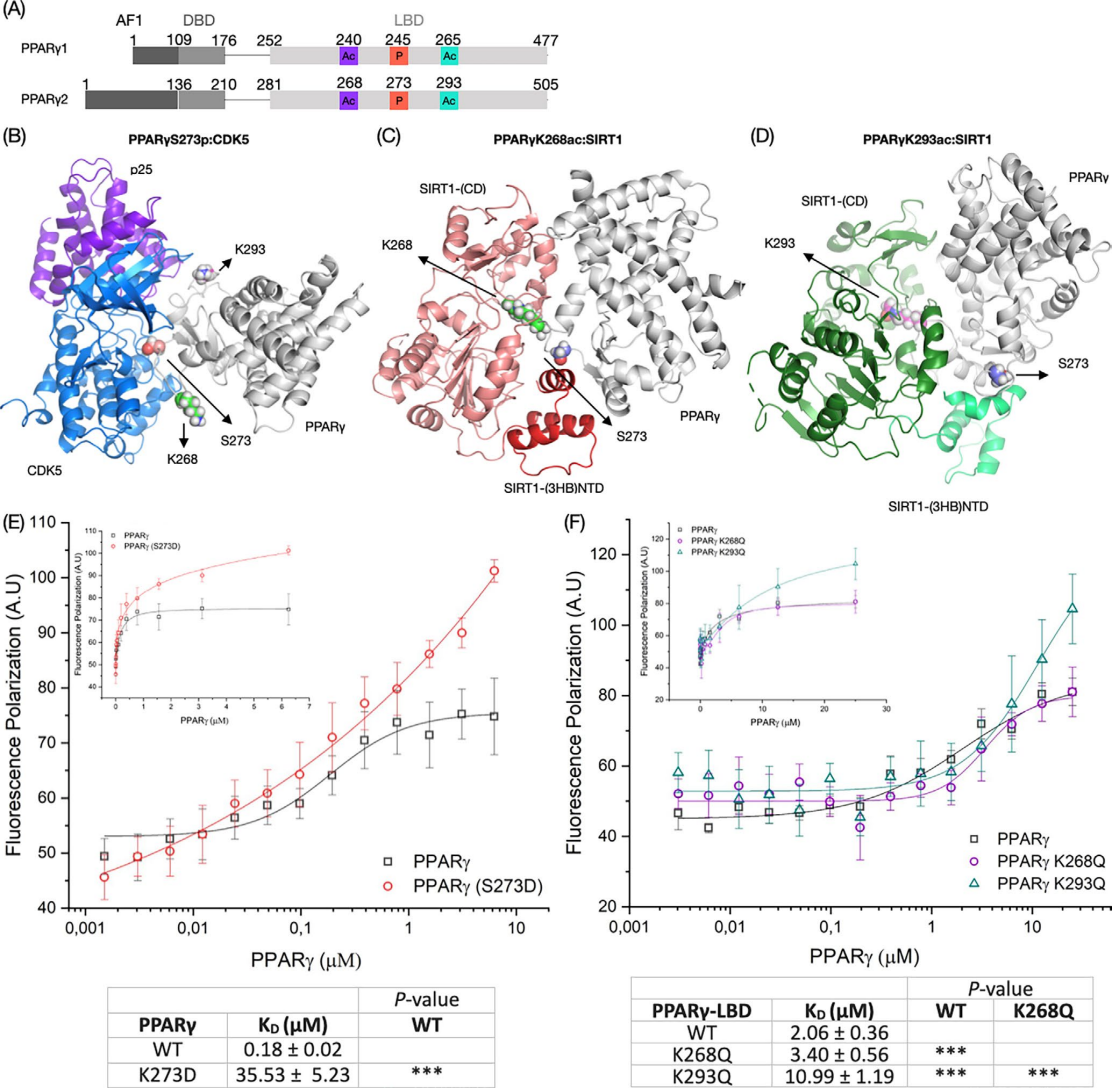
Structural insights into PPARγ‐regulator interactions and the impact of PTMs. (A) Schematic representation of PPARγ isoforms highlighting the position of PTMs within LBD domain. (B) CDK5:PPARγ structural model showing key interaction residues. PPARγ (LBD) (gray) docks onto CDK5 (blue), with S273 (red spheres) positioned in the catalytic cleft, and K268 (green spheres) and K293 (blue spheres) serving as anchoring points. (C) SIRT1:PPARγ model with PPARγ‐K268ac (green spheres). SIRT1 CD (salmon) and NTD (light green) interact with PPARγ(LBD) (gray); S273 is highlighted in blue spheres. (D) SIRT1:PPARγ model with PPARγ‐K293ac (red spheres), showing similar interactions as in (C). S273 (blue spheres) and both SIRT1 domains are indicated. FP binding assays of (E) SIRT1:PPARγ and (F) CDK5:PPARγ carrying phospho‐ and acetyl‐ mimetic mutations, respectively. FP values are plotted as a function of concentration gradient, and *K*_D_ values are shown in the table below each graph. Data represent mean ± SEM, and statistical significance is indicated as follows: *p ≤ 0.05, **p ≤ 0.01, ***p ≤ 0.001, ****p ≤ 0.001.

This study explores how PTMs of PPARγ, specifically K268 acetylation (K268ac), S273 phosphorylation (S273p), and K293 acetylation (K293ac), influence its regulation by the proteins SIRT1 and CDK5. Although PPARγ exists as two major isoforms (PPARγ1 and PPARγ2) that differ only in their N‐terminal region, their LBDs are identical. Throughout this work, we use the PPARγ2 numbering convention. Accordingly, the PTMs examined here occur at K268, S273, and K293 in PPARγ2, corresponding to K240, S245, and K265 in PPARγ1 (Figure 1A). While the residue numbering differs between isoforms, these sites occupy the same structural positions within the shared LBD. Functionally, these modifications are embedded within a regulatory network that critically influences insulin sensitivity. Deacetylation at K268 and K293 by SIRT1 is associated with improved insulin responsiveness, whereas phosphorylation at S273 by CDK5 is linked to the development of insulin resistance.

The initial in silico CDK5:PPARγ models show that CDK5 engages PPARγ through a composite interface involving the β‐sheet region, the H2–H2′ loop containing the phosphorylation site S273, and the flexible H2′–H3 loop. These regions form a combination of hydrophobic contacts and electrostatic interactions that position PPARγ correctly for recognition and phosphorylation by CDK5 [21]. In other words, SIRT1 engages PPARγ through a composite interface formed by its N‐terminal (NTD) and catalytic domain (CD), which together clamp the PPARγ LBD. In both K268ac and K293ac models, the acetylated lysines position near the SIRT1 catalytic site and the NTD consistently anchors to the same region of PPARγ, defining a shared docking platform. Differences arise from loop‐specific contacts highlighting how distinct acetylation sites subtly reshape the binding interface [12].

To experimentally quantify the impact of these PTMs on protein–protein interactions, we employed FP to quantify the dissociation constants (*K*_D_) between SIRT1 or CDK5 and PPARγ (LBD) variants carrying phospho‐ or acetyl‐mimetic mutations. As shown in Figure 1E, SIRT1 exhibited a binding affinity to wild‐type (WT) PPARγ (LBD) in the nanomolar range(*K*_D_ = 0.18 ± 0.02 μM), whereas the S273D mutant showed a markedly reduced affinity to micromolar range (*K*_D_ = 35.53 ± 5.23 μM), indicating that phosphorylation at S273 significantly impairs SIRT1:PPARγ complex formation. We next evaluated the impact of PPARγ K268ac and K293ac on CDK5 binding. As shown in Figure 1F, WT PPARγ (LBD) bound CDK5 with a *K*_D_ of 2.06 ± 0.36 μM. The K268Q acetyl‐mimetic mutant exhibited a moderate reduction in affinity (*K*_D_ = 3.40 ± 0.56 μM), while the K293Q mutant showed a more substantial loss of binding (*K*_D_ = 10.99 ± 1.19 μM). These results suggest that acetylation at K293 strongly impairs CDK5 binding, while phosphorylation at S273 significantly disrupts SIRT1 interaction—highlighting the regulatory importance of these residues in PPARγ (LBD) complex formation. Therefore, chemical modifications at residues K268, S273, and K293, located within the binding interface of PPARγ with its regulatory proteins, alter the interaction dynamics with CDK5 and SIRT1.

### 3.2 PPARγ Transcriptional Activity Is Modulated Through PTMs

PPARγ activation is influenced by the overexpression of CDK5 and SIRT1, suggesting that alterations in cellular phosphorylation and acetylation states can modulate its transcriptional activity [9, 18, 19, 40]. Given that CDK5 overexpression is commonly associated with insulin‐resistant conditions, we aimed to investigate whether acetylation at lysines 268 and 293 affects PPARγ activity under elevated CDK5 levels. To this end, we employed a cell‐based luciferase reporter assay to assess the impact of acetyl‐mimetic mutations (K268Q and K293Q) on PPARγ transactivation in the context of CDK5 overexpression. In this assay, luciferase activity serves as a direct readout of PPARγ‐mediated transcription. Since transactivation reflects PPARγ's capacity to initiate transcriptional programs, structural changes induced by acetylation at these residues could influence its interaction with coactivators, corepressors, or regulatory kinases—ultimately altering the transcriptional response to upstream modulators such as CDK5.

**FIGURE 2:**
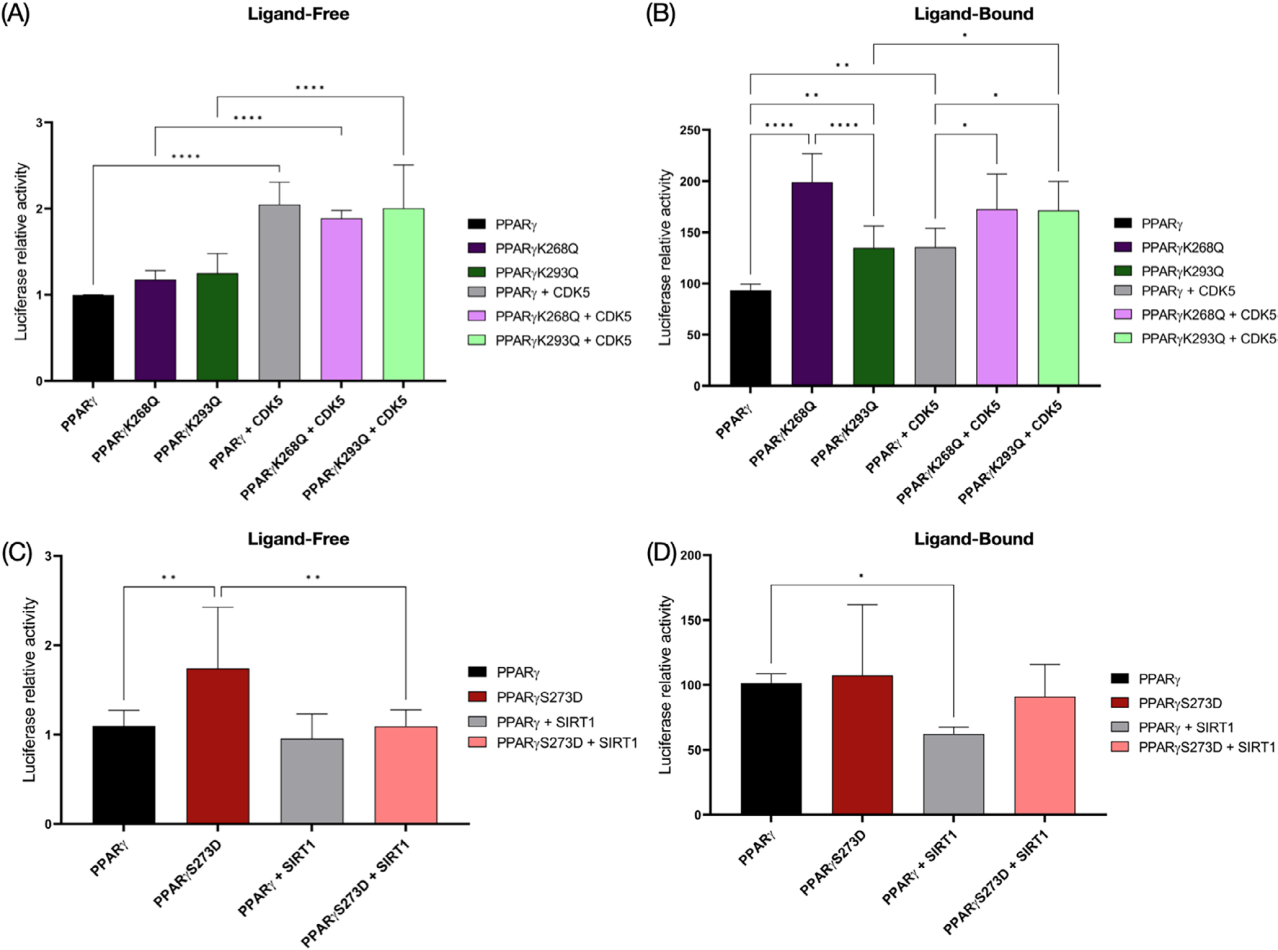
The PMTs and SIRT1 and CDK5 overexpression modulate PPARγ activity. Luciferase reporter assays were performed in 293 T cells co‐transfected with PPARγ wild‐type (WT) or acetyl‐mimetic mutants (K268Q and K293Q), with or without CDK5 in under (A) ligand‐free (DMSO) or (B) ligand‐bound (rosiglitazone) conditions, and for PPARγ WT and S273D, with or without SIRT1 co‐expression under (C) ligand‐free (DMSO) or (D) ligand‐bound (rosiglitazone) conditions. Data represent mean ± SEM and statistical significance: *p ≤ 0.05, **p ≤ 0.01, ***p ≤ 0.001, ****p ≤ 0.0001.

Figure 2A shows the trans‐activation values of PPARγ in the ligand‐free state, representing its repressive conformation. The introduction of acetyl‐mimetic mutations at positions K268 and K293 did not alter the transcriptional activity of PPARγ compared to the WT, indicating that these modifications alone do not modulate PPARγ activation. Interestingly, overexpression of CDK5 led to a significant increase in PPARγ activity in the WT context, suggesting that CDK5 counteracts the repressive state of PPARγ and promotes transcriptional activation [6]. To test whether acetylation at K268 or K293 influences this effect, we evaluated CDK5 overexpression in the acetyl‐mimetic mutants. Both K268Q and K293Q mutants displayed similar trans‐activation levels as WT PPARγ when co‐expressed with CDK5, indicating that acetylation in the ligand‐free state does not interfere with CDK5's effect on PPARγ repression.

Next, we examined these conditions under ligand‐bound activation, as shown in Figure 2B, which represents the active conformation of PPARγ. Under these conditions, both K268Q and K293Q mutants exhibited increased transcriptional activity compared to WT, with K268Q showing the most pronounced effect. This suggests that acetylation at K268 may enhance PPARγ's transcriptional potential in its active state, possibly by stabilizing interactions within the transcriptional machinery. CDK5 overexpression also increased PPARγ activation to a similar extent as the K293Q mutant. When CDK5 was overexpressed in combination with either acetyl‐mimetic mutant, no additive effect was observed. Notably, K268Q co‐expressed with CDK5 displayed trans‐activation values comparable to K268Q alone; reinforcing the idea, that acetylation at K268 may mimic or occlude the effects of CDK5. Together, these results suggest that K268 acetylation may play a role in modulating the pathway of CDK5‐mediated activation of PPARγ, particularly in its ligand‐bound, active form.

The overexpression of SIRT1 leads to the deacetylation of PPARγ K268ac and K293ac and has been associated with insulin‐sensitivity outcomes [18, 19, 40]. We have shown that S273D reduces the binding affinity of PPARγ for SIRT1; therefore, we hypothesize that the effects of SIRT1 overexpression on PPARγ activity would be modulated by the presence of S273p. To test this, we determined the PPARγ transcriptional activity using the PPARγ trans activation assay; for that, we generated a phospho‐mimetic mutant (S273D) and assessed its transcriptional activity using a cell‐based luciferase reporter assay.

We first analyzed the effects of a phosphomimetic mutation on PPARγ transcriptional activity. In the ligand‐free state, the S273D mutant exhibited increased transcriptional activation compared to WT, suggesting that phosphorylation at S273 may disrupt the repressive conformation of PPARγ, thereby enhancing its activity (Figure 2C). Interestingly, overexpression of SIRT1 in this context did not significantly alter the transcriptional activity of either wild‐type or S273D PPARγ, suggesting that the SIRT1 overexpression mutation may counteract the activation effect of the phosphomimetic mutation under these conditions. In the ligand‐bound (active) state, the S273D mutant displayed activation levels like WT PPARγ (Figure 2D). However, overexpression of SIRT1 led to a marked reduction in transcriptional activity, reinforcing the notion that SIRT1 acts as a repressor when PPARγ is in its active conformation. Notably, the combination of S273D and SIRT1 overexpression resulted in activity levels comparable to wild‐type PPARγ in the absence of SIRT1. This suggests a potential trend in which the S273D mutation attenuates the inhibitory effect exerted by SIRT1, revealing a functional interplay between phosphorylation at S273 and SIRT1‐mediated regulation.

### 3.3 Interplay Between Acetylation and Phosphorylation on PPARγ

**FIGURE 3:**
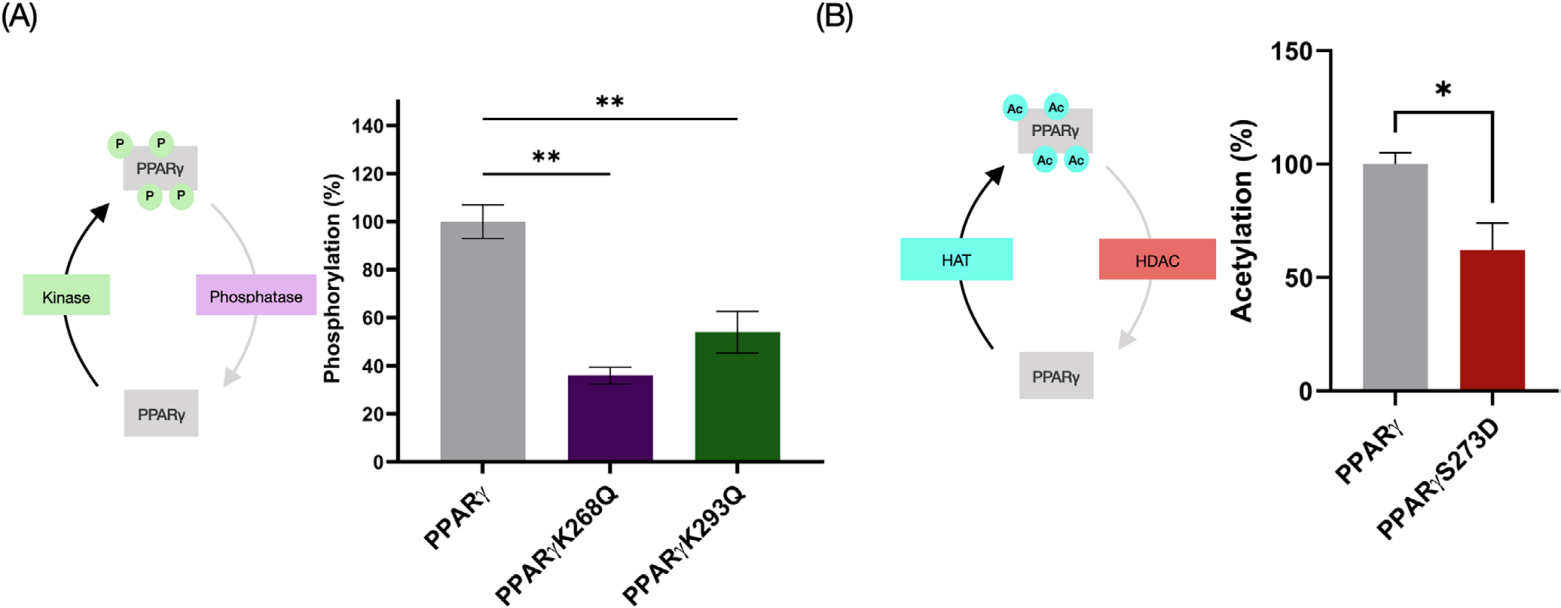
The balance of phosphorylation and acetylation is shifted due to K268ac, S273p, and K293ac. (A) Phosphorylation and (B) acetylation levels of PPARγ(LBD) through in vitro assays. A schematic representation shows the regulation of phosphorylation levels (A) and acetylation levels (B), HAT, histone‐acetyl transferase, and HDAC, histone deacetylase, by regulatory proteins. Data represent mean ± SEM and statistical significance is indicated as follows: *p ≤ 0.05, **p ≤ 0.01, ***p ≤ 0.001.

Given the observed weakening of protein–protein interactions between PPARγ carrying PTMs and the regulatory protein SIRT1 and CDK5, and their influence on PPARγ transcriptional activity, we next investigated whether such modifications influence the balance of acetylation and phosphorylation levels of PPARγ. The homeostasis of PPARγ PTMs depends on the coordinated activity of modifying enzymes, and disruption at one site may impact modification status at others. To assess this potential crosstalk, we performed in vitro acetylation assays using the PPARγ(LBD) phospho‐mimetic S273D mutant and in vitro phosphorylation assays using the PPARγ(LBD) acetyl‐mimetic K268Q and K293Q mutants. As shown in Figure 3A, phosphorylation levels were significantly reduced in both K268Q and K293Q mutants compared to wild‐type PPARγ. Similarly, Figure 3B shows that acetylation was significantly lower in the S273D mutant relative to the wild type. These results indicate that PTMs at K268, K293, and S273 can interfere with the accessibility or activity of modifying enzymes at other sites, supporting the existence of a regulatory interplay between acetylation and phosphorylation in PPARγ.

### 3.4 Computational Assessment of Post‐Translational Modulation of PPARγ Interactions

Building upon our previous FP assays, which indicated a weakened interaction between PPARγ and the regulatory proteins in the presence of acetyl‐ and phosphor‐mimetic mutations, we employed computational simulations to further understand the energetic and structural consequences of these PTMs. We conduct MD simulations to gain insight about the stability of the CDK5:PPARyp and SIRT1:PPARyac complex carrying K268ac and K293ac, and K273p, respectively. The SIRT1:PPARyac complex shows stability over the time course of MD simulation (Figure S1A). However, S273p seems to affect the interface NTD‐PPARy, mainly when K268 is bound to the active site of SIRT1, when fractional native contacts of the interfaces (Q) and distances between SIRT1 domains and PPARy (Figures S2A and S4A). The S273p shows that some replicas affect the SIRT1(NTD)‐PPARy interface for SIRT1:PPARyK293 (Figure S2). In the CDK5:PPARγac simulations, the WT complex (gray lines) maintained a relatively stable RMSD throughout the 1 μs trajectory (Figure S1B), while two replicas explored alternative binding modes, leading to variations at the CDK5:p25:PPARy interface (Figure S3). Besides the conformational changes observed by RMSD and Q analysis (Figures S1B and S3), dissociation was not observed for any replica (Figure S5). In contrast, all replicas of the K293ac complex (green lines) exhibited an increase in RMSD in relation to WT. For K293ac complex one replica showed partial dissociation (Figure S5). Notably, acetylation at K268ac (purple lines) caused a sustained and more pronounced RMSD increase across all replicas, suggesting progressive destabilization of the complex two replicas showed dissociation—(Figures S3 and S5).

To further explore the stability of the complexes, we performed MM–PBSA analysis [41]. For the analysis, different internal dielectric constants (ε_in_) were scanned (1, 2, 4, 6, and 8), converging in ε_in_ ≥ 4. In the case of the SIRT1:PPARγK268ac model, the introduction of S273 phosphorylation appeared to decrease the calculated ΔG, whereas for the SIRT1:PPARγK293ac models, one of the conformations (Model 2) showed an apparent increase in ΔG values. These findings suggest that acetylation and phosphorylation may modulate the energetic landscape of the complex. However, the observed effects were modest and not consistently reproduced across replicates and therefore should be interpreted with caution (Figure S6A and Table S1). In contrast, for the CDK5:PPARγp complex, the introduction of K268ac and K293ac led to a reproducible increase in ΔG in the double mutant, consistent with reduced complex stability. This trend agrees with the experimental observations from FP assays (Figure S6B and Table S2).

**FIGURE 4:**
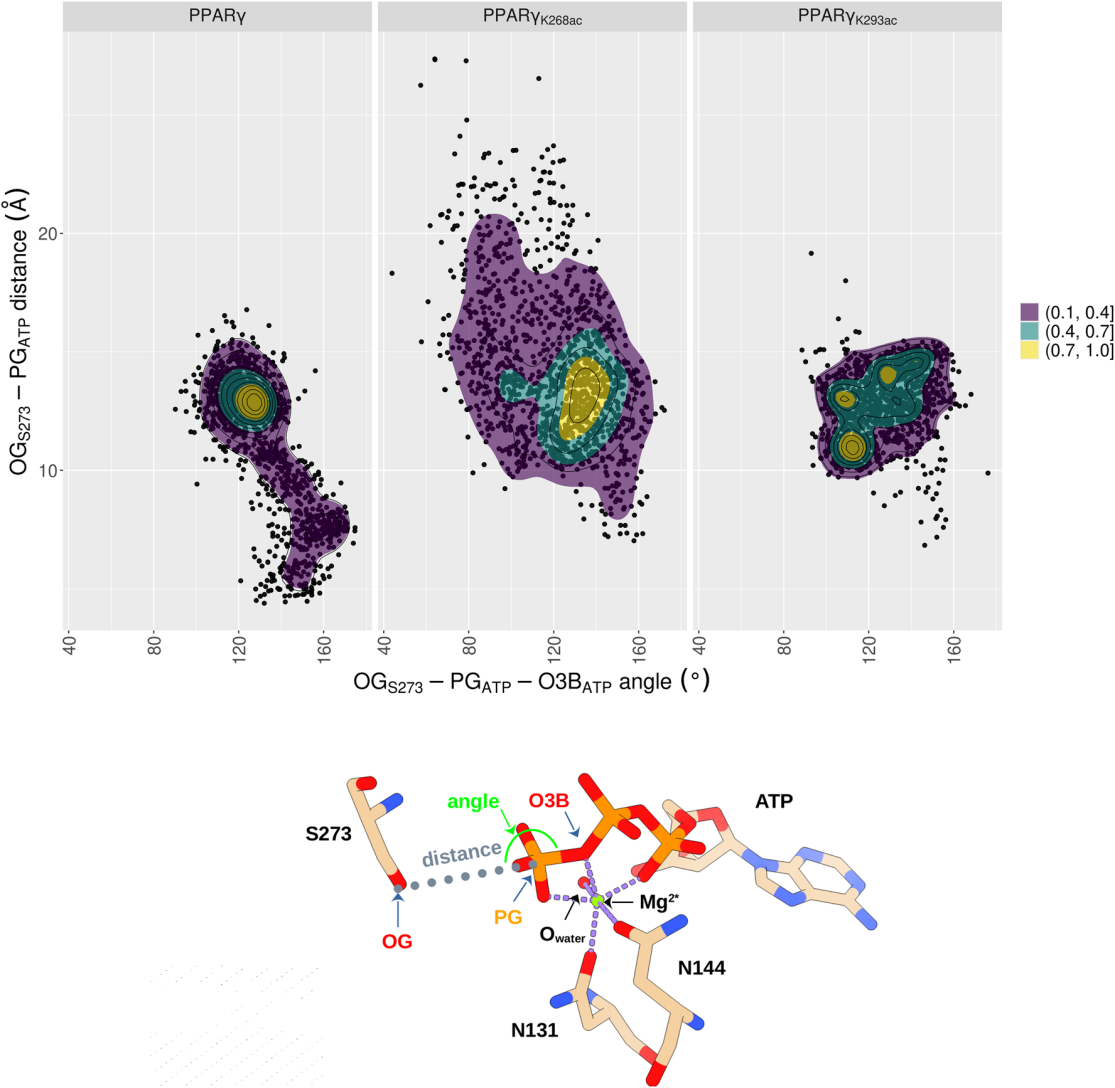
Analysis of S273 positioning for phosphorylation during MD simulations. Top: 2D density maps representing the distribution of OγS273–PγATP distances and OγS273–PγATP–O3βATP angles for WT, K268ac, and K293ac mutants. The data were taken from the three 1 μs MD replicas for each system. The color gradient represents normalized conformational density, with brighter colors indicating more frequently sampled distance–angle combinations during the simulation. Down: Structural representation of the ATP‐binding pocket, with key catalytic residues and ATP coordination shown.

Building upon our in vitro phosphorylation assays, which demonstrated that acetylation reduces CDK5 activity, our in silico analysis further supports this observation at the structural level. Acetylation impacts the reactive configurations involved in phosphate transfer, particularly the spatial arrangement between the catalytic residues and the ATP phosphate group. The Oγ_S273_–Pγ_ATP_ distance and the Oγ_S273_–Pγ_ATP_–O3β_ATP_ angle can be two metrics to analyze the potential reactivity of a configuration in the CDK5–PPARγ. The optimal distance and angle are < 4.9 Å for reactions having an associated character and longer for those with a dissociative character. In addition, the angle should be near to 180° [42]. For example, in the case of CDK2, the crystallographic distance is 3.68 Å [43, 44, 45, 46, 47]. Figure 4 presents the two‐dimensional distribution of the Oγ_S273_–Pγ_ATP_ distances and the Oγ_S273_–Pγ_ATP_–O3β_ATP_ angles sampled throughout the MD trajectories. The WT complex exhibited a higher probability of adopting geometries compatible with effective phosphate transfer, whereas the acetyl‐mimetic models showed a shift toward non‐productive conformations, characterized by increased distances and suboptimal angles. These data suggest that acetylation at K268 and K293 reduces the conformational flexibility necessary for efficient CDK5‐mediated phosphorylation of PPARγ, potentially impairing downstream signaling.

## 4 Discussion

Lysine acetylation at K268 and K293 and phosphorylation at S273 are key PTMs of PPARγ involved in glucose homeostasis. These residues are located at the binding interface between PPARγ and its regulatory partners SIRT1 and CDK5. SIRT1‐mediated deacetylation improves insulin sensitivity, while CDK5‐driven phosphorylation promotes insulin resistance [9, 10, 40]. We tested whether modifications at this interface modulate binding dynamics with SIRT1 and CDK5, as well as PPARγ phosphorylation and acetylation patterns. These findings support PTM‐targeted strategies for modulating PPARγ activity beyond ligand‐based approaches.

We employed PPARγ mimetic variants (K268Q, K293Q, and S273D) to approximate the effects of acetylation and phosphorylation. These substitutions reproduce the major electrostatic consequences of their respective PTMs but do not fully capture their chemical or structural features. Glutamine neutralizes the positive charge of lysine but lacks the planar acetyl group, whereas aspartic acid introduces a negative charge yet is considerably smaller than phosphate. Accordingly, these mimetics provide insight into charge‐related and conformational influences but should not be regarded as exact biochemical equivalents. We also overexpressed SIRT1 and CDK5 to evaluate how shifts in cellular acetylation and phosphorylation states modulate PPARγ activity. Although overexpression does not match endogenous protein levels, it enables controlled mechanistic investigation, and the responses observed align with the established physiological roles of these enzymes. Finally, MM–PBSA calculations were used to qualitatively compare binding energetics; given the end‐state nature of this method and its sensitivity to dielectric parameters, the resulting ΔG values are interpreted as relative trends rather than absolute measurements [41].

**FIGURE 5:**
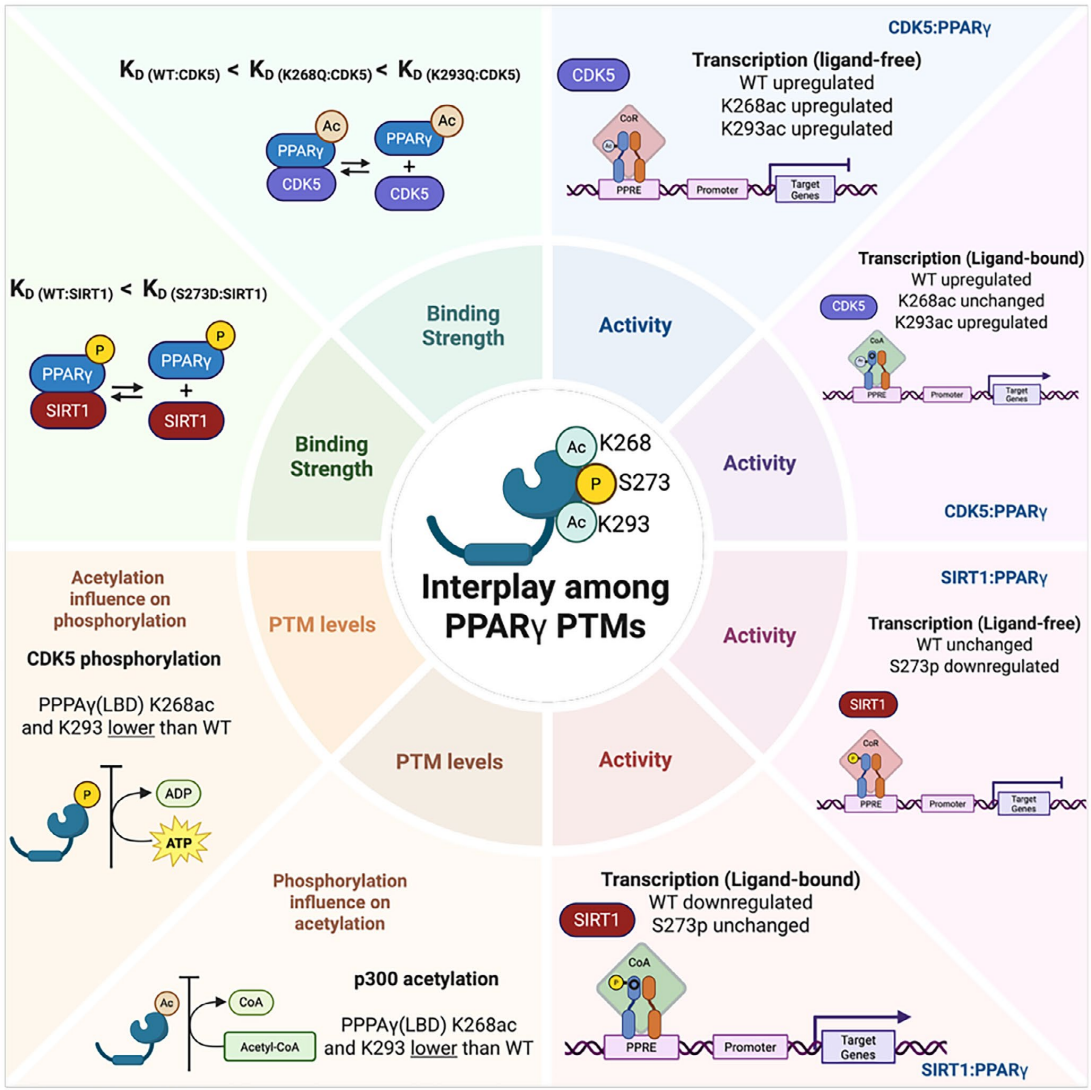
Interplay among PPARγ post‐translational modifications (PTMs) and their impact on protein interactions and transcriptional regulation. Schematic summary integrating the experimental and computational findings of this study. (Top left) Binding affinity measurements show that CDK5 interaction with PPARγ is progressively weakened upon acetylation at K268 and K293, while SIRT1 binding is reduced by S273 phosphorylation. (Bottom left) PTM crosstalk analysis indicates that PPARγ acetylation (K268ac, K293ac) decreases CDK5‐mediated phosphorylation, whereas S273 phosphorylation reduces p300‐mediated acetylation. (Right panels) Functional transcriptional assays reveal context‐dependent modulation: Functional transcriptional assays reveal context‐dependent modulation: In CDK5:PPARγ complexes, K268ac, and K293ac alter ligand‐free and ligand‐bound transcriptional activity, but K268ac has a different outcome than WT e K293ac when CDK5 is overexpressed; in SIRT1:PPARγ complexes, SIRT1 overexpression inhibits activation, but the S273p is able to rescue activation in the ligand‐bound state. Together, these results highlight the dynamic and reciprocal regulation of PPARγ activity through PTMs, shaping both binding interfaces and transcriptional outcomes. Together, these results highlight the dynamic and reciprocal regulation of PPARγ activity through PTMs, shaping both binding interfaces and transcriptional outcomes. Created with BioRender.com.

We then evaluate how K268ac and K293ac affect CDK5 binding, enzymatic activity, and PPARγ transcriptional output under conditions of CDK5 overexpression (Figure 5). Structural modeling identified these residues as critical for CDK5 docking, suggesting that modifications at this binding interface could modulate the interaction dynamics [21]. Supporting this, acetyl‐mimetic mutations (K268Q, K293Q) increased complex instability in MD simulations and reduced both CDK5 binding and CDK5‐mediated PPARγ phosphorylation in vitro—reinforcing the functional relevance of this region as a CDK5 docking site and validating our molecular model. Additionally, mutations at K268 and K293 positions have already been described to decrease CDK5 binding to the receptor; therefore, they collaborate with earlier findings [21, 45]. Functionally, CDK5 overexpression enhanced PPARγ activity in the absence of ligand in agreement with previous findings [6], and the K268Q and K293Q mutants showed similar or increased activation in the ligand‐bound state. This suggests that either enhanced CDK5‐mediated phosphorylation or modifications within the K268/K293 region may influence PPARγ interactions with co‐regulatory proteins [6]. Integrating these findings, we observed that CDK5 overexpression had no additional effect on PPARγ activity in the K268Q mutant, which likely reflects impaired CDK5 binding and reduced kinase activity toward PPARγ. Notably, CDK5 overexpression may alter the global phosphorylation landscape, disrupting cellular homeostasis and indirectly modulating PPARγ function. Altogether, these results indicate that acetylation at K268 and K293 may attenuate CDK5 binding while favoring transcriptional activation—potentially through enhanced coactivator recruitment or shifts in PPARγ phosphorylation patterns [6].

Next, we investigated how S273p influences SIRT1 binding, PPARγ transcriptional output under SIRT1 overexpression, and whether this modification affects global acetylation levels of PPARγ (Figure 5). Structural analysis of the SIRT1:PPARγ complex revealed that S273 lies within the binding interface, specifically interacting with NTD(3HB) of SIRT1—an interface previously identified as a common docking site across multiple binding models [12]. Indeed, PPARγ S273D shows weaker binding strength with SIRT1, demonstrating that S273 lies on the binding interface with SIRT1 in agreement with the molecular models. However, MD simulation of the complex showed global stability profiles based on RMSD values, suggesting no major conformational disruption detectable by these metrics. This could be due to limitations in the timescale or resolution of the simulation in capturing more subtle or transient destabilizations caused by phosphorylation. We next investigated whether phosphorylation at S273 affects the acetylation status of PPARγ. We found that the S273D mutant displayed reduced global acetylation levels, possibly reflecting impaired HAT activity (p300) toward PPARγ. This indicates that S273 is in the binding interface of HAT and may influence its function within the cell. Functionally, S273D increases PPARγ activation and overexpression of SIRT1 in the ligand‐free state, and SIRT1 represses PPARγ activation in the ligand‐bound state. SIRT1 has been shown to recruit corepressor complexes (e.g., NCoR) to inhibit PPARγ activity [15] and may explain the repressive outcomes of SIRT1 overexpression. In the ligand‐bound state, the S273D along with SIRT1 overexpression did not statically differ from solely SIRT1 present. However, it has the same values as WT, which may indicate a trend of S273D to inhibit the inhibition of PPARγ activity due to SIRT1 overexpression. This suggests that phosphorylation at S273 can modulate SIRT1's repressive effects, possibly by disrupting the interaction required for corepressor recruitment.

Taken together, these findings highlight the complexity of PPARγ regulation in metabolic disorders such as insulin resistance, where modulation of transcriptional programs contributes to disease progression. While potent PPARγ agonists can restore insulin sensitivity, their clinical use is limited by side effects, underscoring the need for more targeted strategies that fine‐tune, rather than fully activate, PPARγ activity. In obesity, high levels of S273 phosphorylation shift PPARγ's transcriptional profile without increasing its overall activity [48]. In this context, our data show that acetylation at K268 and K293 does not markedly oppose CDK5‐driven transcriptional effects. Instead, S273 phosphorylation impairs SIRT1 binding, reducing repression and preserving PPARγ activity even under SIRT1 overexpression. This suggests that promoting deacetylation via SIRT1 may be less effective when S273 phosphorylation is elevated, as in obesity. Also, the region encompassing K268, S273, and K293 serves as a critical binding interface for the docking and activity of regulatory proteins, directly influencing PPARγ's transcriptional function. This highlights a promising therapeutic avenue: targeting this interface with molecules that enhance K268ac and K293ac deacetylation while weakening CDK5 interaction. In support of this concept at the cellular level, glucose uptake assays in mature adipocytes showed that TNFα significantly reduced glucose uptake, whereas subsequent treatment with resveratrol partially restored this effect (Figure S7). Moreover, resveratrol pretreatment prevented the inhibitory impact of TNFα, indicating a protective role of SIRT1 activation and demonstrating that modulation of the cellular acetylation state can counteract CDK5‐driven insulin resistance. By modulating these specific PTMs and disrupting detrimental protein–protein interactions, it may be possible to selectively adjust PPARγ activity without triggering the broad activation typically associated with agonist‐based therapies. Such an approach could improve insulin sensitivity with fewer side effects, offering a more refined strategy for treating metabolic disorders like insulin resistance.

Altogether, our findings demonstrate that fine‐tuning PTMs at the PPARγ regulatory interface represents a viable alternative to full receptor activation. This interface‐centric approach opens new avenues for selectively modulating PPARγ activity in metabolic diseases, with the potential for improved therapeutic precision and reduced adverse effects.

## Author Contributions

**Caique Camargo Malospirito:** investigation, conceptualization, methodology, formal analysis, data curation, writing – review and editing, formal analysis. **Marieli Mariano Gonçalves Dias:** investigation, conceptualization, methodology, formal analysis, data curation. **Gabriel Ernesto Jara:** methodology, investigation, writing – review and editing, formal analysis, software. **Thayná Mendonça Avelino:** investigation, methodology, validation. **Giovanna Blazutti Elias:** investigation, methodology, validation. **Paulo Sergio Lopes de Oliveira:** formal analysis, data curation. **Ana Carolina Migliorini Figueira:** conceptualization, funding, data curation, formal analysis, writing – review and editing.

## Funding

This research was funded by the São Paulo Research Foundation (FAPESP) for funding this work (Grant Numbers: #2019/10274‐7, #2022/05028‐0, and #2022/11624‐4).

## Conflicts of Interest

The authors declare no conflicts of interest.

## Supporting information


**Data S1:** Supporting Information.
**Figure S1:** MDs of CDK5 and SIRT1 interactions with acetyl‐ and phosphorylated PPARγ variants. (A) RMSD of three 1000 ns MD replicas for SIRT1:PPARγ complexes, showing that the phosphorylated PPARγ variants. The backbone of the catalytic domain of SIRT1 was used for alignment. The systems are: SIRT1:PPARγ when either K268 (SIRT1:PPARγK268) or K293 (SIRT1:PPARγK293 Model 2) bound to the SIRT1 active site with either PPARγ native (gray scale lines) or phosphorylated at S273 (S273p, (red scale lines)). (B) RMSD of three 1000 ns MD replicas for CDK5:PPARγ complexes. The backbone of the CDK5 was used for alignment. The systems shown are: CDK5 in complex with PPARγ without acetylation, acetylated in K268 (K268ac), and acetylated in K293 (K293ac). The plots on the right show the distribution of RMSD. Three replicas were run for each system (labeled as R1, R2, or R3). For all cases, the RMSD was calculated using the protein backbone.
**Figure S2:** Fractions of native contact (Q) for SIRT1:PPARγ. The systems are: SIRT1:PPAR γ when either K268 (SIRT1:PPARγK268) or K293 (SIRT1:PPARγK293 Model 2) bound to the SIRT1 active site with either PPARγ native (gray scale lines) or phosphorylated at S273 (S273p, red scale lines). (A) The interface between PPARγ and the catalytic domain of SIRT1. (B) The interface between PPARγ and the NTD of SIRT1. The plots on the right show the distribution of fractions of native contacts (Q). Three replicas were run for each system (labeled as R1, R2, or R3).
**Figure S3:** Fractions of native contact (Q) for CDK5:p25:PPARγp model carrying K268ac and K293ac. The systems show are: CDK5:PPARγp (black lines) and carrying K268ac (purple) and L293ac (cyan). Three replicas were run for each system (labeled as R1, R2, or R3).
**Figure S4:** Distance of the center of mass between SIRT:PPARγ models. The models SIRT1:PPARγK268 and SIRT1:PPARγK293 (Model 2) display SIRT1 active site bound to PPARγac at K268 and K293, respectively. The native SIRT1:PPARγ (gray scale lines) or phosphorylated at S273 (S273p, red scale lines). (A) The distance between the centers of mass of NTD domain of SIRT and PPARγ. (B) The distance between the centers of mass of the catalytic domain of SIRT and PPARγ. The center of mass was calculated using the protein backbone. For the center of mass of PPARγ, H12 was not considered because of its high flexibility. Three replicas were run for each system (labeled as R1, R2, or R3).
**Figure S5:** Distance of the center of mass between CDK5:p25 and PPARγ. The systems shown are: CDK5 in complex with PPARγ without acetylation, PPARγK268ac, and PPARγK293ac. The center of mass was calculated using the protein backbone. For the center of mass of PPARγ, H12 was not considered because of its high flexibility. Three replicas were run for each system (labeled as R1, R2, or R3).
**Figure S6:** MM‐GBSA analysis of CDK5 and SIRT1 interactions with acetyl‐mimetic and phosphorylated PPARγ variants. (A) MM‐GBSA ΔG values for SIRT1 in complex with native and acetylated PPARγ variants, with (gray) or without (magenta) phosphorylation at S273. Phosphorylation at S273 consistently reduces binding affinity. (B) Binding free energy (ΔG) estimates calculated via MM‐GBSA for CDK5 in complex with native PPARγ (gray), PPARγ:K268ac (magenta), and PPARγ:K293ac (green) across a range of internal dielectric constants. Acetylation at K268 or K293 enhances the predicted stability of the complex. *p ≤ 0.05, **p ≤ 0.01, ***p ≤ 0.001, ****p ≤ 0.0001.
**Figure S7:** Glucose uptake modulation by ligands in adiposity. Three T3‐L1 cells differentiated into adipocytes and treated with ligands modulate SIRT1 and CDK5 pathways. (A) TNF‐ α induces phosphorylation via CDK5; (B) Resveratrol induces deacetylation via SIRT1. Statistical analysis: One‐way ANOVA. *p ≤ 0.05, **p ≤ 0.01, ***p ≤ 0.001, ****p ≤ 0.0001.
**Table S1:** MM/PBSA results for the SIRT1:PPAy complex, showing the mean binding free energy (ΔG mean) and the corresponding lower (95% CI lower bound) and upper (95% CI upper bound) confidence interval bounds estimated via bootstrap (kcal·mol−1).
**Table S2:** MM/PBSA results for the CDK5/PPAy complex, showing the mean binding free energy (ΔG mean) and the corresponding lower (95% CI lower bound) and upper (95% CI upper bound) confidence interval bounds estimated via bootstrap (kcal·mol−1).

## Data Availability

The data that support the findings of this study are available on request from the corresponding author. The data are not publicly available due to privacy or ethical restrictions.
