# Supplementary material for: Interplay of Ser273 Phosphorylation and K268 and K293 Acetylation in PPARγ: Implications for PPARγ Activation
Source: Proteins. 2026 Apr 12;94(9):1597–609. doi: 10.1002/prot.70140 (PMC13435072; doi:10.1002/prot.70140)
Supplement: Supplementary file 1 [file PROT-94-1597-s001.pdf]

Interplay of Ser273 Phosphorylation and K268 and K293 Acetylation in PPARγ: Implications for PPARγ activation

Modulation of PPARγ Activity via Post-Translational Crosstalk

Caique Camargo Malospirito[1,2], Marieli Mariano Gonçalves Dias[1,2], Gabriel Ernesto Jaha[1], Thayná Mendonça Avelino[1], Giovanna Blazutti Elias[1,4], Paulo Sergio Lopes de Oliveira[1], Ana Carolina Migliorini Figueira[1*]

[1]Brazilian Center for Research in Energy and Materials (CNPEM), Brazilian Biosciences National Laboratory (LNBio), Campinas, Brazil.

[2]Graduate Program in Molecular and Morphofunctional Biology, Institute of Biology, State University of Campinas (Unicamp), Campinas, Brazil.

[4]Graduate Program in Pharmaceutical Sciences, Faculty of Pharmaceutical Sciences, State University of Campinas (Unicamp), Campinas, Brazil.

Corresponding author: ana.figueira@lnbio.cnpem.br

Supplementary Results

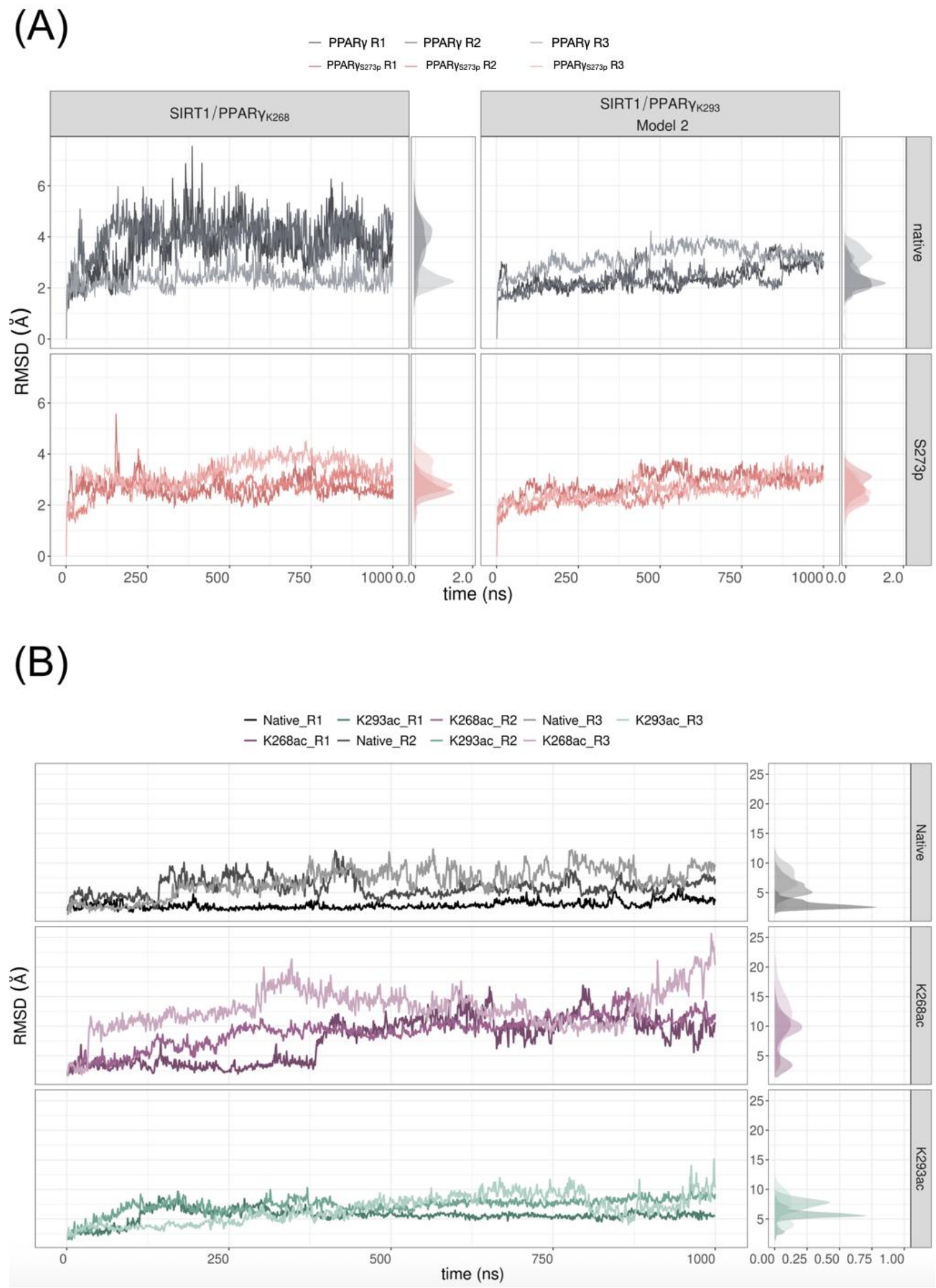

Supplementary Figure S1. MDs of CDK5 and SIRT1 interactions with acetyl- and phosphorylated PPARγ variants. (A) RMSD of three 1000 ns MD replicas for SIRT1:PPARγ complexes, showing that the phosphorylated PPARγ variants. The backbone of the catalytic domain of SIRT1 was used for alignment. The systems are: SIRT1:PPARγ when either K268 (SIRT1:PPARγ$_{K268}$) or K293 (SIRT1:PPARγ$_{K293}$ Model 2) bound to the SIRT1 active site with either PPARγ native (gray scale lines) or phosphorylated at S273 (S273p, (red scale lines)). (B) RMSD of three 1000 ns MD replicas for CDK5:PPARγ complexes. The backbone of the CDK5 was used for alignment . The systems show are: CDK5 in complex with PPARγ without acetylation, acetylated in K268 (K268ac) and acetylated in K293 (K293ac). The plots on the right show the distribution of RMSD. Three replicas were run for each system (labelled as R1, R2 or R3). For all cases, the RMSD was calculated using the protein backbone.

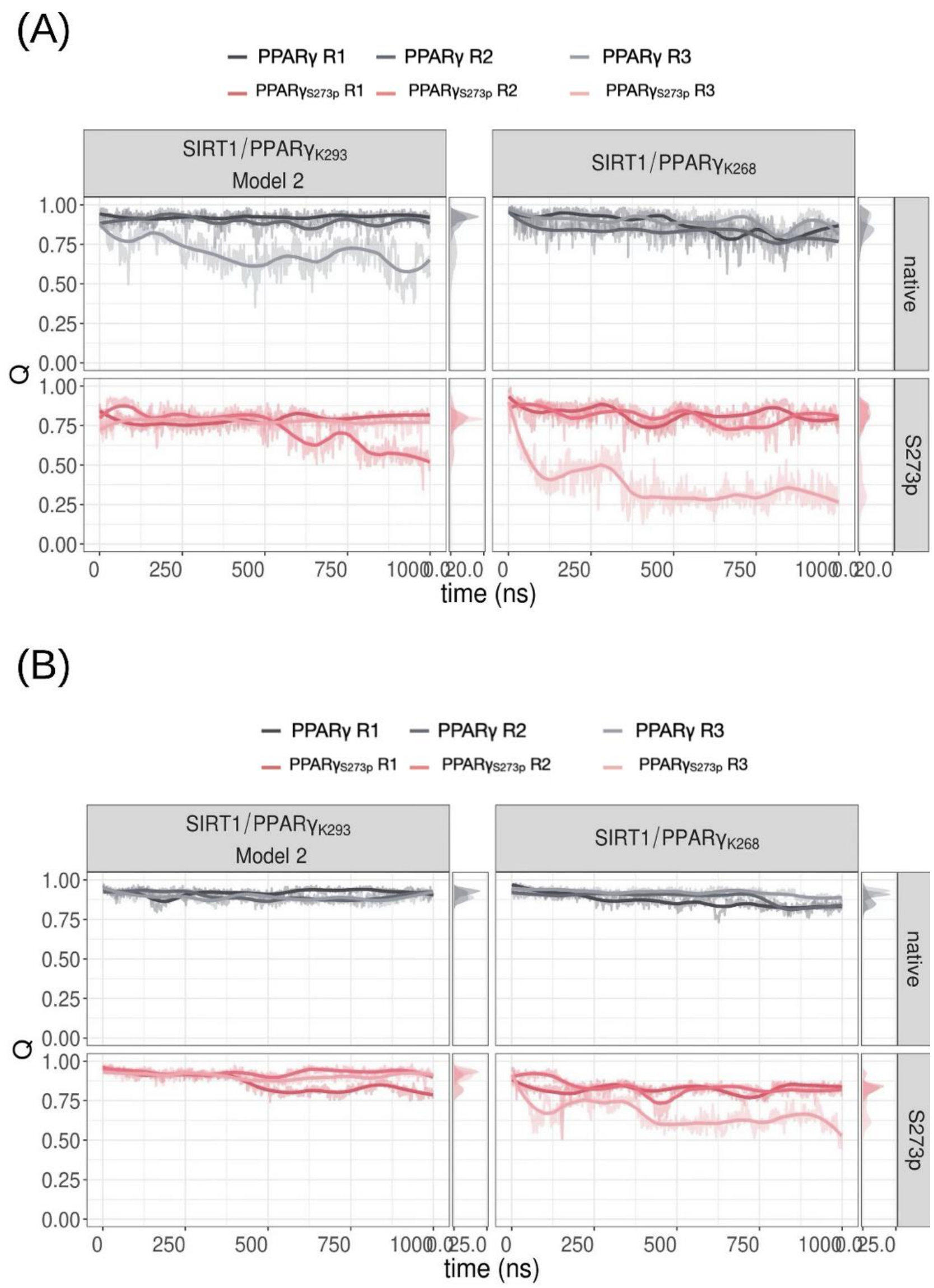

Supplementary Figure S2. Fractions of native contact (Q) for SIRT1:PPAR γ. The systems are: SIRT1:PPAR γ when either K268 (SIRT1:PPAR γ_{K268}) or K293 (SIRT1:PPAR γ_{K293} Model 2) bound to the SIRT1 active site with either PPAR γ native (gray scale lines) or phosphorylated at S273 (S273p, red scale lines). (A) The interface between PPAR γ and the catalytic domain of SIRT1. (B) The interface between PPAR γ and the NTD of SIRT1. The plots on the right show the distribution of fractions of native contacts (Q). Three replicas were run for each system (labelled as R1, R2 or R3).

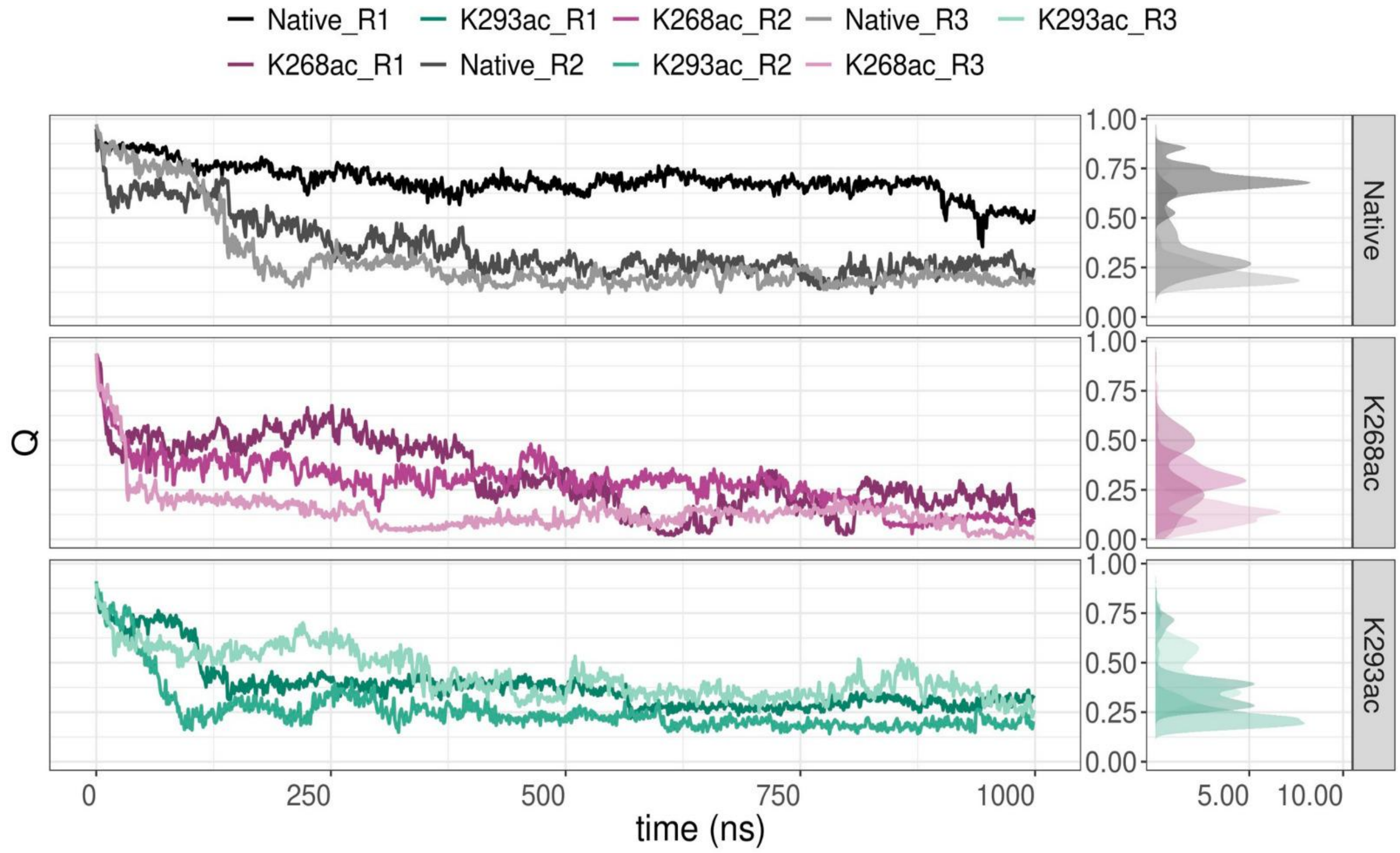

Supplementary Figure S3. Fractions of native contact (Q) for CDK5:p25:PPAR γ p model carrying K268ac and K293ac. The systems show are: CDK5:PPARγp (black lines) and carrying K268ac (purple) and L293ac (cyan) . Three replicas were run for each system (labelled as R1, R2 or R3).

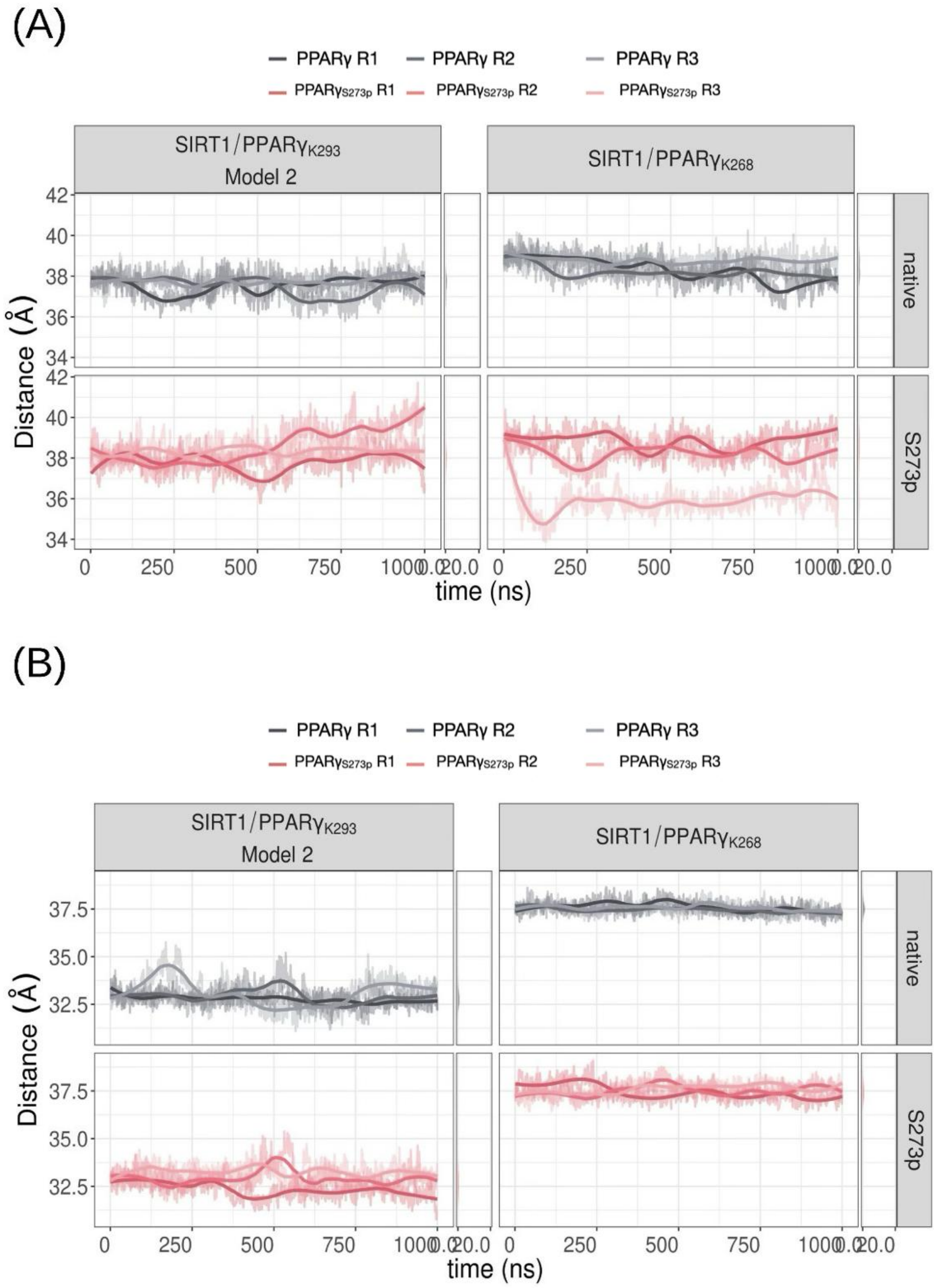

Supplementary Figure S4. Distance of the center of mass between SIRT:PPAR γ models. The models SIRT1:PPAR γ $_{K268}$ and SIRT1:PPAR γ $_{K293}$ (Model 2) display SIRT1 active site bound to PPAR γ ac at K268 and K293 respectively. The native SIRT1:PPAR γ (gray scale lines) or phosphorylated at S273 (S273p, red scale lines). (A) The distance between both centers of mass of NTD domain of SIRT and PPAR γ . (B) The distance between both centers of mass of catalytic domain of SIRT and PPAR γ . The center of mass was calculated using the protein backbone. For the center of mass of PPARγ, H12 was not consider because of its high flexibility. Three replicas were run for each system (labelled as R1, R2 or R3).

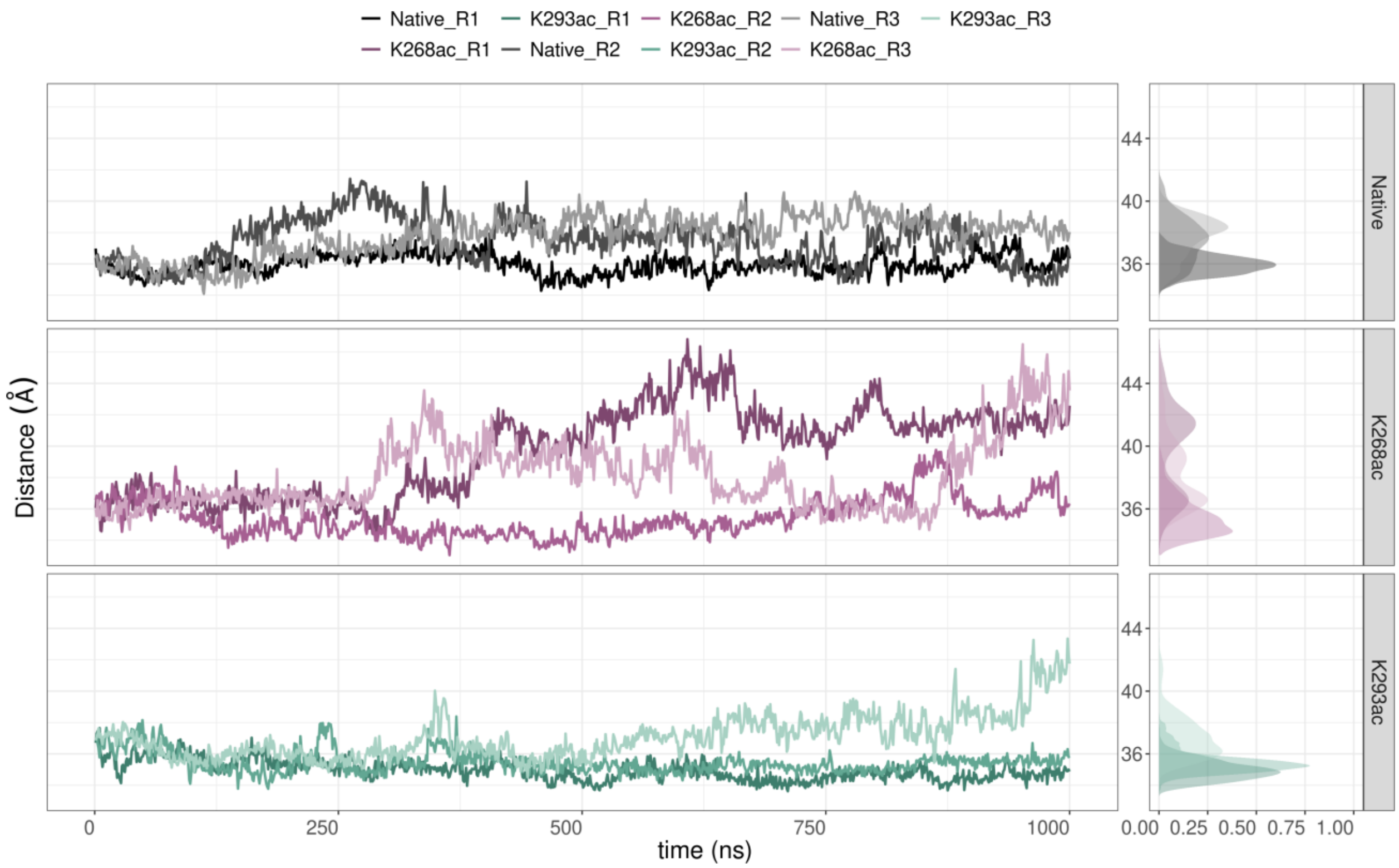

Supplementary Figure S5. Distance of the center of mass between CDK5:p25 and PPARγ. The systems show are: CDK5 in complex with PPARγ without acetylation, PPARγK268ac and PPARγK293ac. The center of mass was calculated using the protein backbone. For the center of mass of PPARγ, H12 was not consider because of its high flexibility. Three replicas were run for each system (labelled as R1, R2 or R3).

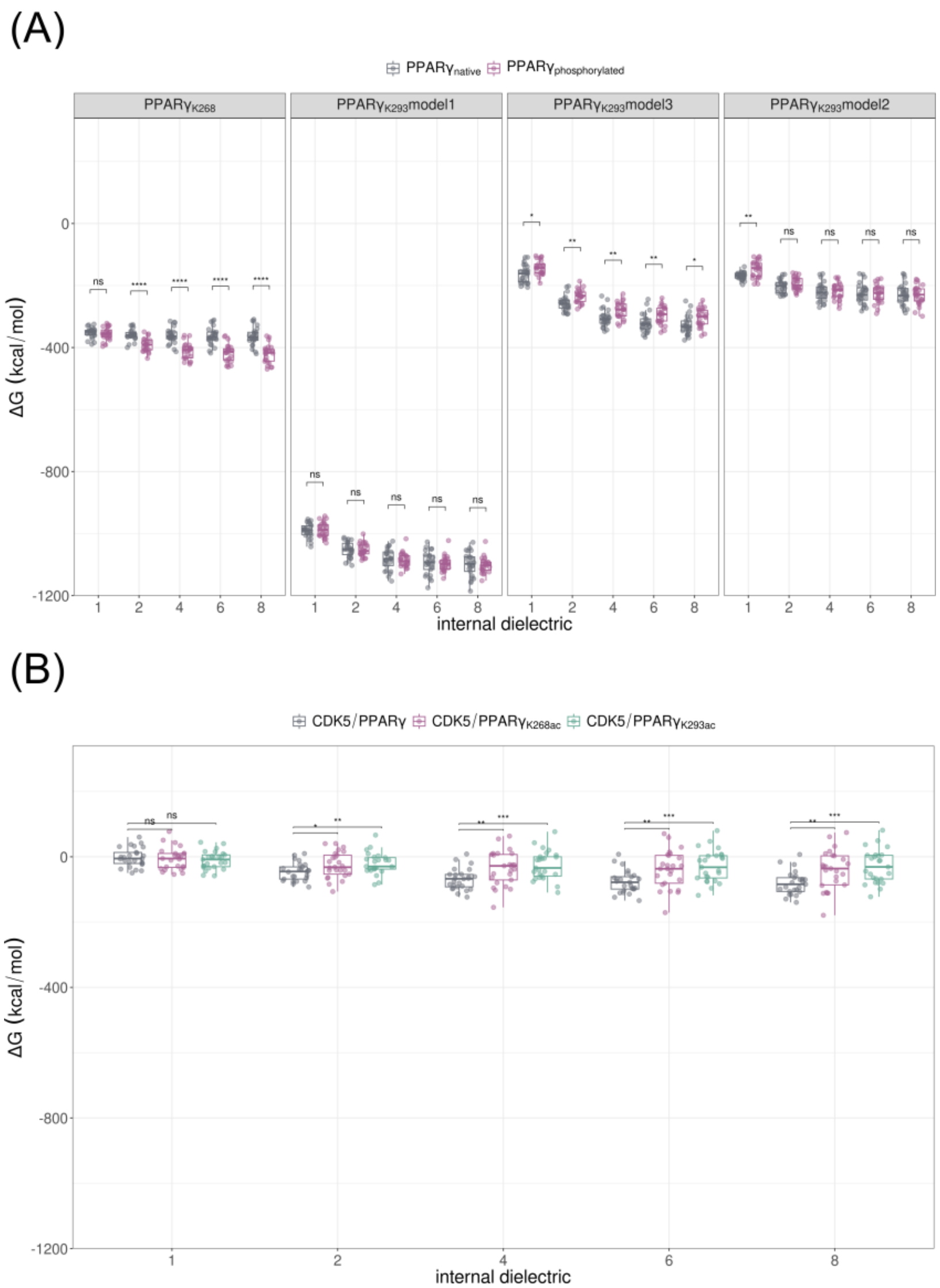

Supplementary Figure S6. MM-GBSA analysis of CDK5 and SIRT1 interactions with acetyl-mimetic and phosphorylated PPARγ variants. (A) MM-GBSA ΔG values for SIRT1 in complex with native and acetylated PPARγ variants, with (gray) or without (magenta) phosphorylation at S273. Phosphorylation at S273 consistently reduces binding affinity. (B) Binding free energy (ΔG) estimates calculated via MM-GBSA for CDK5 in complex with native PPARγ (grey), PPARγ:K268ac (magenta), and PPARγ:K293ac (green) across a range of internal dielectric constants. Acetylation at K268 or K293 enhances the predicted stability of the complex. $^{*}p \leq 0.05$, $^{**}p \leq 0.01$, $^{***}p \leq 0.001$, $^{****}p \leq 0.0001$

Glucose Uptake

Methods:

Given the regulatory role of SIRT1 and CDK5 in metabolic signaling, we assessed glucose uptake in differentiated adipocytes to determine whether their modulation impacts cellular glucose handling. For that, Pre-adipocyte 3T3-L1 cells were cultured in complete DMEM containing 10% FBS and 1% (w/v) penicillin/streptomycin. For differentiation, 15,000 cells per well were seeded in 96-well plates with 100 µL of complete DMEM and maintained at 37 °C until they reached 100% confluence. Once confluent, the medium was replaced with a Differentiation Induction Medium consisting of DMEM with 10% FBS, 1% (w/v) antibiotic, 1 µM dexamethasone, 1 µg/mL insulin, and 0.5 mM IBMX (3-isobutyl-1-methylxanthine). Cells were incubated in this medium for 48 h at 37 °C. Following induction, the medium was changed to a Maintenance Medium composed of DMEM with 10% FBS, 1% (w/v) antibiotic, and 1 µg/mL insulin. This medium was refreshed every 48 h for a total of 14 days to allow complete adipocyte differentiation. After adipocyte differentiation, cells were subjected to the glucose uptake assay. The differentiation medium was replaced with EMEM without serum supplemented with 2.5 nM TNFα. After 24 h, this medium was replaced with serum-free EMEM, and cells were incubated for 1 h at 37 °C. Subsequently, the medium was changed to high-glucose DMEM containing the first set of treatments (2.5 nM TNFα or 10 µM resveratrol). After 1 h, the second treatment was added to the appropriate conditions to generate the combined TNFα and resveratrol group. One hour later, the culture medium was collected, and glucose concentration was quantified using a colorimetric Glucose GOD-PAP assay (Enzymax), following the manufacturer's instructions. Data were analyzed in GraphPad Prism using one-way ANOVA followed by Tukey's post hoc test. Statistical significance was defined as. Data analysis was performed with GraphPad Prism software, using one-way ANOVA and Tukey's test, with $*p \leq 0.05$, $**p \leq 0.01$, $***p \leq 0.001$, $****p \leq 0.0001$

Results:

TNF α is a pro-inflammatory cytokine known to activate CDK5, increase cellular phosphorylation levels, and contribute to an insulin-resistant phenotype[1–3]. Conversely, resveratrol is a polyphenol that promotes insulin sensitivity by activating SIRT1 and shifting the cellular acetylation balance. To assess the functional implications of the CDK5 and SIRT1 pathways, we treated mature adipocytes with TNF α and/or resveratrol and measured glucose uptake (Figure S2). When adipocytes were first exposed to TNF α and subsequently treated with resveratrol, TNF α markedly reduced glucose uptake, whereas the following resveratrol treatment partially restored it. This indicates that the TNF α-induced impairment of insulin

sensitivity can be only partially overridden by SIRT1 activation, suggesting that CDK5-driven phosphorylation alone is insufficient to fully counteract the effects of resveratrol. Conversely, when resveratrol pretreatment preceded TNF α exposure, the stimulatory effect on glucose uptake was largely maintained despite the presence of TNF α. These results suggest that resveratrol confers a protective, insulin-sensitizing effect that limits TNF α -mediated disruption of glucose metabolism.

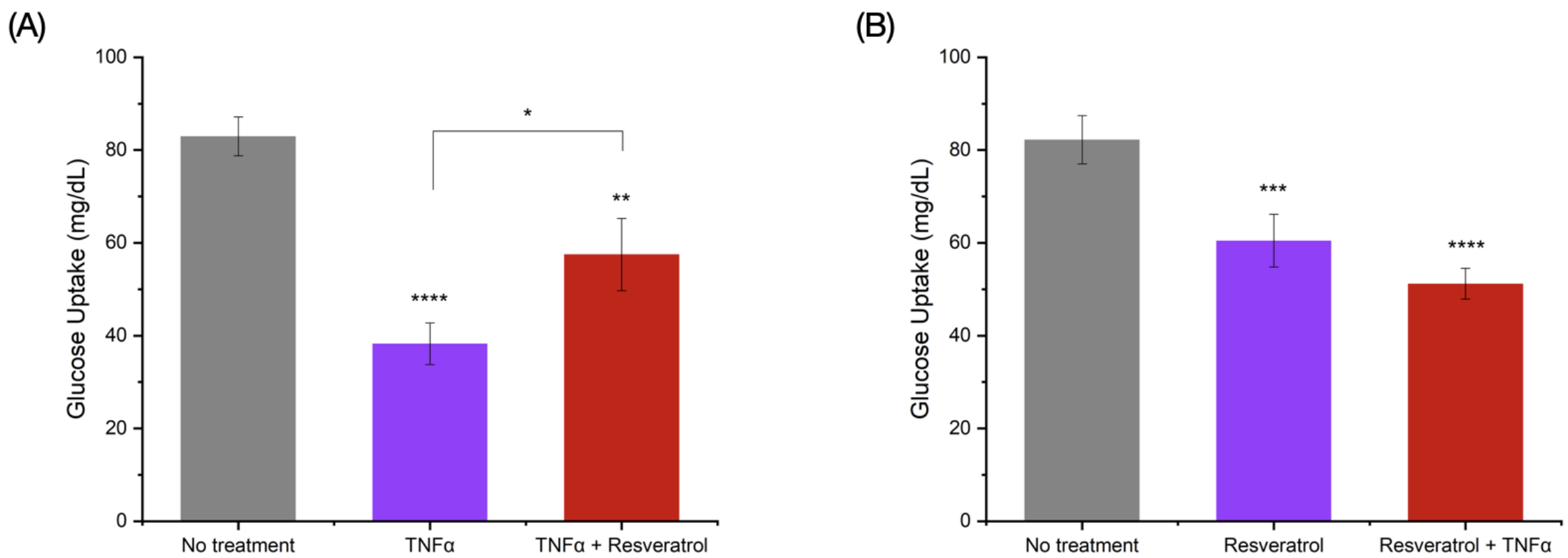

Supplementary Figure S7. Glucose uptake modulation by ligands in adipocity. 3T3-L1 cells differentiated into adipocytes and treated with ligands modulates SIRT1 and CDK5 pathways. (A) TNF-α induces phosphorylation via CDK5; (B) Resveratrol induces deacetylation via SIRT1. Statistical analysis: One-way ANOVA. *$p \leq 0.05$, **$p \leq 0.01$, ***$p \leq 0.001$, ****$p \leq 0.0001$

Supplementary Table S1. MM/PBSA results for the SIRT1:PPAγ complex, showing the mean binding free energy (ΔG mean) and the corresponding lower (95% CI lower bound) and upper (95% CI upper bound) confidence interval bounds estimated via bootstrap (kcal·mol^{-1}).

system	replica	Mean ΔG	95% CI lower bound	95% CI upper bound
SIRT1:PPARγ$_{K268}$	1	-353.79	-358.87	-348.88
	2	-375.89	-382.94	-368.92
	4	-386.67	-396.36	-377.00
	6	-390.30	-400.98	-379.53
	8	-392.24	-403.35	-381.12
SIRT1:PPARγ$_{K293\ model1}$	1	-989.19	-995.52	-982.92
	2	-1052.39	-1058.65	-1046.12
	4	-1084.75	-1092.61	-1076.80
	6	-1095.82	-1104.38	-1087.30
	8	-1101.34	-1110.23	-1092.45
SIRT1:PPARγ$_{K293\ model3}$	1	-152.86	-160.43	-145.33
	2	-245.99	-252.89	-239.03
	4	-292.74	-300.77	-284.58
	6	-308.36	-316.93	-299.65
	8	-316.25	-325.08	-307.27
SIRT1:PPARγ$_{K293\ model2}$	1	-158.30	-164.65	-151.69
	2	-198.83	-204.68	-192.85
	4	-219.73	-227.42	-212.03
	6	-226.99	-235.39	-218.55
	8	-230.67	-239.50	-221.79

Supplementary Table S2. MM/PBSA results for the CDK5/PPAy complex, showing the mean binding free energy (ΔG mean) and the corresponding lower (95% CI lower bound) and upper (95% CI upper bound) confidence interval bounds estimated via bootstrap (kcal·mol^{-1}).

system	label	Mean ΔG	95% CI lower bound	95% CI upper bound
CDK5:PPARγ	1	-2.12	-13.05	9.21
	2	-46.36	-56.71	-35.61
	4	-69.09	-81.26	-56.15
	6	-76.96	-89.77	-63.31
	8	-81.05	-94.41	-66.94
CDK5:PPARγ[K268ac]	1	-6.20	-18.74	7.05
	2	-25.59	-40.75	-10.47
	4	-35.63	-55.82	-15.77
	6	-39.05	-61.11	-17.13
	8	-40.83	-64.01	-18.16
CDK5:PPARγ[K293ac]	1	-10.56	-20.91	0.17
	2	-20.57	-33.05	-7.54
	4	-26.10	-43.00	-8.77
	6	-28.24	-46.82	-9.25
	8	-29.49	-48.89	-9.73

References

1. Avelino, T. M. *et al.* Improving obesity research: Unveiling metabolic pathways through a 3D In vitro model of adipocytes using 3T3-L1 cells. *PLoS One* 19, e0303612 (2024).
2. Cao, D. *et al.* Structural basis for allosteric, substrate dependent stimulation of SIRT1 activity by resveratrol. *Genes Dev* 29, 1316–1325 (2015).
3. Qiang, L. *et al.* Brown remodeling of white adipose tissue by SirT1-dependent deacetylation of Pparγ. *Cell* 150, 620–632 (2012).
